# Infections with *Gyrodactylus* spp. (Monogenea) in Romanian fish farms: *Gyrodactylus salaris* Malmberg, 1957 extends its range

**DOI:** 10.1186/s13071-016-1727-7

**Published:** 2016-08-11

**Authors:** Haakon Hansen, Călin-Decebal Cojocaru, Tor Atle Mo

**Affiliations:** 1 Norwegian Veterinary Institute, PO Box 750 Sentrum, N-0106 Oslo, Norway; 2 Sanitary Veterinary and Food Safety Laboratory, 4 Surorile Martir Caceu, 300585 Timisoara, Romania

**Keywords:** Mitochondrial cytochrome *c* oxidase subunit I, *Gyrodactylus salaris*, *Gyrodactylus truttae*, Rainbow trout, *Oncorhynchus mykiss*, *Salvelinus fontinalis*, *Salmo trutta*, Romania

## Abstract

**Background:**

The salmon parasite *Gyrodactylus salaris* Malmberg, 1957 has caused high mortalities in many Atlantic salmon, *Salmo salar*, populations, mainly in Norway. The parasite is also present in several countries across mainland Europe, principally on rainbow trout, *Oncorhynchus mykiss*, where infections do not seem to result in mortalities. There are still European countries where there are potential salmonid hosts for *G. salaris* but where the occurrence of *G. salaris* is unknown, mainly due to lack of investigations and surveillance. *Gyrodactylus salaris* is frequently present on rainbow trout in low numbers and pose a risk of infection to local salmonid populations if these fish are subsequently translocated to new localities.

**Methods:**

Farmed rainbow trout *Oncorhynchus mykiss* (*n* = 340), brook trout, *Salvelinus fontinalis* (*n* = 186), and brown trout, *Salmo trutta* (*n* = 7), and wild brown trout (*n* = 10) from one river in Romania were sampled in 2008 and examined for the presence of *Gyrodactylus* spp. Alltogether 187 specimens of *Gyrodactylus* spp. were recovered from the fish. A subsample of 76 specimens representing the different fish species and localities were subjected to species identification and genetic characterization through sequencing of the ribosomal internal transcribed spacer 2 (ITS2) and mitochondrial cytochrome *c* oxidase subunit 1 (*cox*1).

**Results:**

Two species of *Gyrodactylus* were found, *G. salaris* and *G. truttae* Gläser, 1974. This is the first time *G. salaris* is diagnosed in Romania. *Gyrodactylus salaris* was found to infect rainbow trout, brown trout and brook trout in eight of the 12 farms examined. The prevalence and intensity of infections were generally low in all farms. *Gyrodactylus truttae* was present on brook trout in one farm and on wild brown trout in the river studied. This also represents the first record of this parasite in Romania. Analyses of sequences of the *cox*1 gene of *G. salaris* from Romania revealed four haplotypes, all previously undescribed. While it is not unlikely that the infections in Romanian fish farms originate directly from imported rainbow trout, the current data is not sufficient to conclude on this and does not exclude that the infections can originate from hosts in the local water systems. The study shows that there are still unknown populations and variants (haplotypes) of *G. salaris* present in European rainbow trout aquaculture, all or many of them with unknown biological characteristics such as host specificity and virulence. As some strains might be pathogenic to Atlantic salmon, the importance of carrying out surveillance and keeping a high focus on control with import and export of live fish for aquaculture purposes is important.

**Conclusions:**

*Gyrodactylus salaris* and *G. truttae* are for the first time found on salmonids in Romania. All mitochondrial haplotypes recovered were previously undescribed and this indicates that there is still an unknown diversity of this parasite present in localities not previously examined. The virulence of the haplotypes found in Romania is unknown and requires establishing.

**Electronic supplementary material:**

The online version of this article (doi:10.1186/s13071-016-1727-7) contains supplementary material, which is available to authorized users.

## Background

The monogenean *Gyrodactylus salaris* Malmberg, 1957 causes gyrodactylosis and is responsible for severe epidemics in Atlantic salmon (*Salmo salar* L.) populations in rivers draining in to the North Atlantic Ocean and the White Sea [1]. According to the World Organisation for Animal Health (OIE), infections with *G. salaris* are notifiable (http://www.oie.int/en/animal-health-in-the-world/oie-listed-diseases-2016//) and detection of the parasite can result in trading restrictions for their hosts. Before this study, *G. salaris* had been confirmed to occur in 16 countries (Paladini et al., unpublished data.), and new surveys, as exemplified by the latest from Italy [2] and Poland [3], will likely extend the number of registered countries for this parasite.

*Gyrodactylus salaris* is known to infect a number of host species in addition to its type-host, the Atlantic salmon, although generally without causing disease (see [1] for a review). Both the rainbow trout, *Onchorhyncus mykiss* Walbaum, 1792, and Arctic char, *Salvelinus alpinus* L., seem to be suitable hosts that can sustain infections for long periods [4, 5], but *G. salaris* has also experimentally been shown to live and reproduce on several other fish hosts, such as e.g. the brook trout *Salvelinus fontinalis* Mitchill, 1814, lake trout *Salvelinus namaycush* Walbaum, 1792, grayling *Thymallus thymallus* L., and brown trout *Salmo trutta* L. [1, 6]. In recent years, non-pathogenic strains of *G. salaris* have also been reported [7, 8]. The parasite is particularly common on rainbow trout [2, 3, 9, 10] and due to the risk of introduction to new regions, trade in live susceptible species of listed diseases is only permitted between countries, zones or compartments of equivalent health status (or from higher to lower status) (EU L 328/14).

The currently accepted and applied standard for diagnosis and description of species of *Gyrodactylus* involves DNA sequencing of the ribosomal internal transcribed spacer region (ITS) combined with morphological/morphometric analyses of the haptoral hard parts of the parasite, see e.g. [11–13]. These diagnostic techniques can differ between most morphologically delineated species, but a notable exception to this is the discrimination of *G. salaris* from *G. thymalli* Žitňan, 1960 infecting the grayling [14, 15]. Although morphology as a diagnostic tool alone has been considered adequate for identification of *G. salaris* when performed by trained experts, recent studies, e.g. [16], show that even experts cannot unambiguously differ between *G. salaris* and *G. thymalli*, and thus a morphological diagnosis must today be confirmed by molecular diagnostics, see [13].

Analyses of the mitochondrial cytochrome *c* oxidase subunit 1 gene (*cox*1) sequences of *G. salaris* and *G. thymalli*, although revealing a high degree of genetic variation between samples, do not show monophyly of the two species [10, 17–19] and conspecificity of the species is suggested [10, 17, 19]. A recent study analysing microRNA from a small number of populations of *Gyrodactylus* specimens from *S. salar* and *T. thymallus* [20] presented evidence in support of conspecificity of *G. salaris* and *G. thymalli* and also proposed formal synonymisation. However, the same authors used the name *G. thymalli* for parasites from grayling in a more recent publication [21] and thus it remains to see whether the synonymisation will be accepted by the scientific community. In cases where morphology and ITS sequences from a specimen corresponding to *G. salaris*/*G. thymalli*, and where the *cox*1 sequences cannot be assigned to a previously known haplotype associated with a specific host species, identification today is implicitly host-based. The name *G. thymalli* is thus so far used for parasites from *T. thymallus* only, while specimens from other hosts are named *G. salaris*.

In Romania in 2013, the aquaculture production was 11,007 tonnes and of this, rainbow trout represented only 3000 t [22]. Production of salmonids other than rainbow trout in Romania is negligible, but also brown trout (no details on volume produced available) and brook trout (15 t) is farmed.

So far, 22 species of *Gyrodactylus* have been found in Romania [23–25] including one, *G. derjavini* Mikailov, 1975, on brown trout. Routine surveillance for the presence of *G. salaris* (or other species of *Gyrodactylus*) in fish farms is not implemented in Romania and in general investigations in fish farms are only done on reports of sick fish according to the Romanian national surveillance program (Ord. 29/2014). As the gyrodactylid fauna of Romanian salmonids is not well known, we initiated this study to access their associated gyrodactylid fauna focussing on farmed fish.

## Methods

### Collection of fish

During April, July, August and September 2008, a total of 543 salmonids belonging to three species (*Oncorhynchus mykiss*, *Salmo trutta* and *Salvelinus fontinalis*) were sampled from 12 fish farms and from one river located in the western and central parts of Romania (see Table 1 for sample details). All farms receive intake water from small rivers, except for Văliug-Semenic trout farm (see Table 1), which use groundwater. The intake water in all farms is filtered through sand filters. The fishes sampled in farms were captured in seine nets whilst the fishes sampled from the river were caught by gill net, electrofishing and angling. All fish were killed following the strict codes of practice in force in Europe, before preserving the samples in 96 % ethanol. Small fish were preserved as whole fish, but from larger fish only the fins were excised. No approval from Institutional Animal Care and Use Committee (IACUC) or ethics committee was necessary.

**Table 1:** List of samples analysed (sorted by county and locality/farm); fish hosts, *Gyrodactylus* spp. identified and haplotypes of *G. salaris* found. Different samples from the same locality and date represents different tanks

County/Locality	Date	Fish host	No. of fish examined	Fish size, total length (cm)	No. of *Gyrodactylus* specimens recovered	No. of *Gyrodactylus* specimens analyzed	*Gyrodactylus* spp.	*G. salaris* haplotype
Bihor County								
Chişcău trout farm	14.08.2008	*O. mykiss*	30	20–25	0	0	–	
	14.08.2008	*O. mykiss*	20	10–15	10	5	*G. salaris*	RO2, RO3
	14.08.2008	*S. fontinalis*	10	25–30	10	5	*G. salaris*	RO2, RO3
Vaşcău trout farm	14.08.2008	*O. mykiss*	3	40–60	1	0	not analysed	–
	14.08.2008	*O. mykiss*	50	10–15	10	6	*G. salaris*	RO1, RO2
	14.08.2008	*S. fontinalis*	10	20	0	0	–	–
	14.08.2008	*S. fontinalis*	4	40–50	0	0	–	–
Caraş Severin County								
Miniş trout farm	24.07.2008	*O. mykiss*	30	30	10	5	*G. salaris*	RO1
	24.07.2008	*O. mykiss*	10	10	0	0	–	–
	24.07.2008	*S. fontinalis*	20	30	3	3	*G. salaris*	RO1
Topleţ trout farm	01.09.2008	*O. mykiss*	16	20	10	5	*G. salaris*	RO1
Văliug-Semenic trout farm	24.07.2008	*S. fontinalis*	40	10	1	1	*G. truttae*	–
	24.07.2008	*S. fontinalis*	10	30	0	0	–	–
	24.07.2008	*S. fontinalis*	5	40–60	2	2	*G. salaris*	RO4
Gorj County								
Tismana trout farm	11.04.2008	*O. mykiss*	30	5	10	6	*G. salaris*	RO1, RO2
	11.04.2008	*O. mykiss* ^*a*^	10	5	1	1	*G. salaris*	RO1
	11.04.2008	*S. fontinalis*	20	20	10	5	*G. salaris*	RO1
	31.08.2008	*S. trutta*	1	50	0	0	–	–
	31.08.2008	*O. mykiss*	35	5, 20, 30	20	5	*G. salaris*	RO1
Tismana-Monastery trout farm	11.04.2008	*S. fontinalis*	20	20	10	5	*G. salaris*	RO1
	11.04.2008	*S. fontinalis*	30	5	10	5	*G. salaris*	RO1
	31.08.2008	*S. fontinalis*	17	20–25	31	5	*G. salaris*	RO1
Harghita County								
Bălan trout farm	15.08.2008	*O. mykiss*	20	10–30	0	0	–	–
Lacu Roşu trout farm	15.08.2008	*O. mykiss*	20	10–30	0	0	–	–
Mădăraş trout farm	27.08.2008	*O. mykiss*	6	25–50	2	2	*G. salaris*	RO1
	27.08.2008	*S. trutta*	6	25–50	15	5	*G. salaris*	RO1
Mădăraş-Mădăraşu Mare river	27.08.2008	*S. trutta*	10	20–40	21	5	*G. truttae*	–
Miercurea Ciuc trout farm	15.08.2008	*O. mykiss*	10	30	0	0	–	–
Timiş County								
Româneşti trout farm	18.08.2008	*O. mykiss*	50	6–30	0	0	–	–

Note: RO1 = GQ129460, RO2 = GQ129461, RO3 = GQ129462, RO4 = GQ129463

^a^The farmers had labelled the fish in these tanks as *O. mykiss* hybrid (Kenlop) and they are therefore listed as a separate sample

### Fish examination

Whole fish or fins were placed in boxes filled with 96 % ethanol and examined for the presence of gyrodactylids under a steromicroscope. Parasites were removed from the fish with a micropipette and placed in individual 1.5 ml Eppendorf tubes. When possible, at least ten specimens of *Gyrodactylus* spp. were isolated from each locality (sample) (see Table 1). To try to maximise the number of samples from individual hosts and the genetic diversity studied, parasites were isolated from as many fins as possible, rather than isolating several specimens from the same fin. The rationale behind this is that very often an infection on one fish is the result of a single infection event and that in *Gyrodactylus*, mature specimens give birth to live offspring that will attach close to the mother. Thus, sampling many individuals from the same site on one host often equals sampling individuals of the same clone. In the case of whole fish, usually one specimen was taken from each infected fish. The actual number on each fish was not counted and parasites from each sample were pooled by host. Thus individual assignment of each parasite specimen to individual fish was not possible and exact intensities of infections were not calculated.

### Parasite identification

Species identification in the current study was based on sequencing of the internal transcribed spacer 2 (ITS2) (~450 nt) of the ribosomal rRNA gene cluster. Although the full ITS fragment (consisting of ITS1, 5.8S and ITS2) is recommended by OIE and display greater variation between species, ITS2 alone can discriminate between most known species from salmonids (but see introduction). The ITS2 was thus chosen because the shorter length of the fragment makes amplification easier and more consistent (pers. obs.). Images of the haptoral hard parts and morphometric measurements were taken only to supplement the molecular diagnosis. The specimens were prepared for molecular and morphological analyses according to [2], using the hard elements of the haptoral attachment apparatus for morphometric analyses and the remaining body for molecular analyses, except the haptors were not permanently mounted in ammonium picrate. Instead, in the final step in the digestion procedure the digestion was arrested by adding 2 μl 1:1 formalin:glycerol solution and the mount was sealed with nail varnish.

DNA was extracted from the cut off bodies of individually isolated specimens using the DNEasyKit or Mini Kit (Qiagen, Hilden, Germany) following the manufacturer’s instructions. Between one and six specimens from each sample were chosen randomly from the total sample and subjected to molecular analyses (see Table 1). The primer pair ITS4.5 and ITS2 [26] were used to amplify the ribosomal ITS2 fragment. For further characterisation of a selection of the specimens found to have an ITS2 sequence corresponding to *G. salaris/G. thymalli*, a fragment of the mitochondrial *cox*1 gene was amplified using the primer pair LA and HA [27]. Both PCR reactions were carried out with puRe Taq Ready-to-Go PCR beads (GE Healthcare, Buckinghamshire, UK) in a GeneAmp® PCR System 9700 (Applied Biosystems, Foster City, CA, USA) following previously published PCR protocols for ITS2 [26] and *cox*1 [27].

The PCR-products were purified using a QIAquick PCR Purification Kit (Qiagen) or Macherey-Nagel NucleoSpin® Extract II according to the manufacturer’s recommendations. Both DNA strands were sequenced using the PCR primers on a MEGABACE 1000 (Amersham Biosciences AB, Uppsala, Sweden) using DyeET-terminator mix (GE-Healthcare) or were sent to Macrogen for sequencing. Sequences were proofread in VectorNTI ver. 11.5 (Invitrogen, Carlsbad, USA) and the sequences (full length) were then submitted to a GenBank BlastN search to search for identity with known sequences (http://www.ncbi.nlm.nih.gov/) [28].

All available *cox*1 sequences of *G. salaris* and *G. thymalli* were downloaded from GenBank (as of 08.07.2014) and aligned with the obtained *cox*1 sequences from the current study using Mega 6.0 [29] (see Additional file 1 for a list of sequences). In order to achieve an alignment without missing information, the alignment is based on 745 base pairs of each haplotype (Additional file 2). The alignment of all available haplotypes was then collapsed into a data set containing only unique haplotypes using FABOX [30]. Phylogenetic relationships were then inferred by neighbour-joining in MEGA 6.0 [29] using *G. derjavinoides* (GenBank accession number GQ368225) as the outgroup. Calculations of genetic distances were calculated according to Kimura two-parameter method [31].

The preparations of the digested haptoral hard parts were photographed using a Nikon DXM1200 Digital camera fitted to a Leica DM5000B microscope under a 100× oil immersion objective. The haptoral hooks were then measured using Zeiss AxioVision (Carl Zeiss Vision GmbH, Munchen, Germany) software. The following measurements were used, see [32]: hamulus total length (HTL), hamulus shaft length (HSL), hamulus point length (HPL), hamulus root length (HRL), ventral bar total length (VBTL), ventral bar membrane length (VBMBL), ventral bar total width (VBTW), ventral bar median length (VBML), marginal hook total length (MHTL), marginal hook shaft length (MHSHL), and marginal hook sickle length (MHSL).

## Results

### Gyrodactylid infections and diversity

Specimens of *Gyrodactylus* were found in eight of the 12 farms examined. In total 187 specimens of *Gyrodactylus* were recovered from the three fish species examined. In addition, specimens of *Gyrodactylus* were also found on wild brown trout in the sample from River Mare (Table 1). Due to the fact that, in general, whole fish were not examined, it was impossible to assess the precise number of parasites on each individual host, but there were no indications of high infections on any of the fins. Seventy-six specimens were subjected to PCR and sequencing of the ITS2 fragment; of these, 74 gave positive results and readable sequences. A comparison of the newly-generated ITS2 sequences to sequences in the GenBank database via a BlastN search resulted in a 100 % similarity with sequences for *G. salaris/G. thymalli* (e.g. GenBank accession number AF484544) (*n* = 68) and *G. truttae* (GenBank accession number EF464681) (*n* = 6). No intra-specific variation was found between ITS2 sequences of the same species. The ITS2-sequences (one representative sequence per species) are submitted to GenBank under accession numbers KX423533–KX423534.

**Fig. 1:**
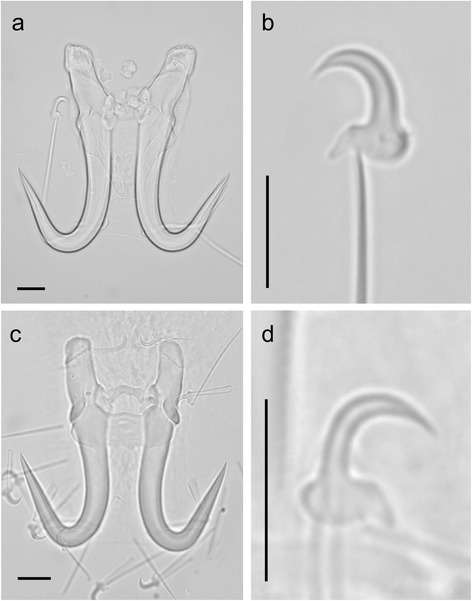
Light micrographs of the haptoral hard parts of *Gyrodactylus salaris* Malmberg, 1957 from *Oncorhynchus mykiss* (**a**, **b**) and *G. truttae* Gläser, 1974 from *Salmo trutta* (**c**, **d**). *Scale-bars*: 10 μm

The morphology (Fig. 1) and measurements (Table 2) of the haptoral hard parts for *G. salaris* and *G. truttae* support the molecular conclusion. Not all microscopic preparations were of sufficient quality for morphometric analyses and thus only 43 specimens of *G. salaris* and five specimens of *G. truttae* were measured. All, except two, measurements for *G. salaris* were within the ranges for measurements previously presented [32–34]: VBMBL (maximum length 23.9 μm in the current study *versus* 23.0 μm previously) and VBTW (maximum length 36.0 μm in the current study *versus* 32.0 μm previously).

**Table 2:** Morphometric measurements (range followed by the mean, in parentheses, all in μm) for specimens of *Gyrodactylus salaris* and *G. truttae* analysed in this study

Structure	*G. salaris* ex *Oncorhynchus mykiss*	*G. salaris* ex *Salvelinus fontinalis*	*G. salaris* ex *Salmo trutta*	*G. truttae* ex *Salvelinus fontinalis*	*G. truttae* ex *Salmo trutta*
	(*n* = 20)	(*n* = 19)	(*n* = 4)	(*n* = 1)	(*n* = 4)
Hamulus total length (HTL)	68.0–78.5 (73.8)	66.9–75.8 (73.2)	73.8–77.2 (75.3)	65.1	65.3–66.0 (65.7)
Hamulus shaft length (HSL)	44.8–50.1 (47.6)	43.8 – 52.0 (48.2)	45.5–49.4 (47.0)	42.0	41.1–42.4 (41.7)
Hamulus point length (HPL)	31.8–42.6 (37.8)	35.0–41.1 (38.0)	37.4–38.1 (37.6)	33.4	33.4–34.2 (33.7)
Hamulus root length (HRL)	25.5–30.4 (27.6)	23.1–29.9 (26.8)	27.8–28.7 (28.3)	21.3	21.2–22.5 (21.8)
Ventral bar total length (VBTL)	26.3–35.1 (31.3)	27.8–33.9 (31.2)	30.8–34.1 (31.7)	31.0	26.8–29.6 (28.1)
Ventral bar membrane length (VBMBL)	13.7–21.8 (17.6)	14.4–23.9 (17.5)	15.6–18.8 (17.2)	19.2	15.1–18.0 (16.4)
Ventral bar total width (VBTW)	26.6–36.0 (31.5)	27.7–35.3 (32.1)	30.0–32.5 (31.0)	31.7	28.5–32.5 (30.8)
Ventral bar median length (VBML)	8.1–13.3 (10.1)	6.5–13.6 (10.5)	8.5–13.6 (10.3)	9.2	8.5 – 10.6 (9.1)
Marginal hook total length (MHTL)	37.8–43.6 (40.4)	36.9–43.8 (40.4)^a^	38.3–41.0 (39.8)	29.4	30.0–30.7 (30.3)
Marginal hook shaft length (MHSHL)	30.5–35.7 (32.7)	29.0–36.1 (33.0)^a^	31.6–33.6 (32.5)	24.7	23.9–24.5 (24.1)
Marginal hook sickle length (MHSL)	7.3 – 8.5 (8.0)	7.2–8.7 (8.0)	7.7–8.1 (8.0)	6.6	6.4–6.7 (6.6)

^a^ Marginal hook total length (MHTL) and Marginal hook sickle length (MHSL) only available from 17 specimens

Two microscopic preparations of the haptoral hard parts for each of the two species are deposited in the Natural History Museum, Oslo, Norway with the following accession numbers: *G. salaris* (NHMO C 6954 and NHMO C 6955) and *G. truttae* (NHMO C 6953 and NHMO C 6956).

### Host specificity

*Gyrodactylus salaris* was found on all three salmonids examined, both in farms where more than one fish host were reared but also on brook trout in two farms where this was the only host present (Table 1). The finding on brown trout occurred in a farm where rainbow trout was also present. *Gyrodactylus salaris* was the most frequently found parasite and was found in all eight positive farms, reflecting the number of suitable hosts examined. *Gyrodactylus truttae* was found on brook trout in one farm and on wild brown trout from the River Mare (Table 1).

### Molecular and phylogenetic analyses of mitochondrial DNA

**Fig. 2:**
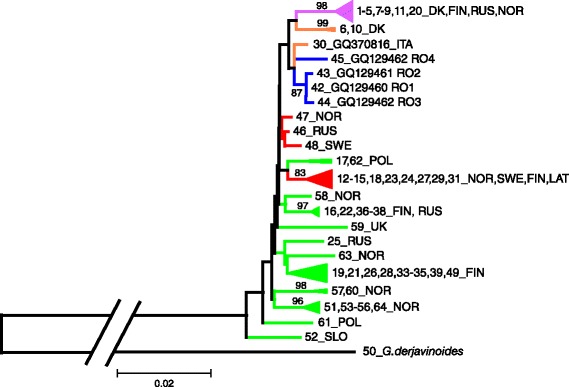
Neighbour-joining tree for 62 mitochondrial *cox*1 haplotypes of *G. salaris* and *G. thymalli* based on a 745 bp alignment. Evolutionary distances were computed using Kimura 2-parameter method. Bootstrap support is indicated as percentages of 1000 replicates; only bootstrap values > 80 % are given. Scale-bar refers to a genetic distance of 0.02. Haplotype number and country of origin are listed beside each branch. Details of the haplotypes included can be found in Additional file 1. *Key*: *Blue* branches: haplotypes from Romania (RO1-4) found on three different hosts (see text); *green* branches: haplotypes from *Thymallus thymallus*; *red* branches: haplotypes from *Salmo salar*; *orange* branches: haplotypes from *Oncorhynchus mykiss*; *pink* branches: haplotypes from *Salmo salar*, *Salmo letnica* and *O. mykiss*. The tree was rooted with *G. derjavinoides. Abbreviations*: DK, Denmark; FIN, Finland; ITA, Italy; LAT, Latvia; NOR, Norway; POL, Poland; RUS, Russia; SLO, Slovakia; SWE, Sweden

PCR and subsequent DNA sequencing of *cox*1 was performed on 31 specimens identified as *G. salaris* by ITS2. Sequences, 854 nt long, were obtained from all 31 specimens and from these, four different haplotypes, labelled RO1-4, were recovered (Table 1.). The BlastN search (based on the full length sequences as of 03.07.14), alignment, and subsequent phylogenetic analyses show that all these haplotypes are new to science (Fig. 2). The newly-generated *cox*1 sequences are submitted to GenBank under accession numbers GQ129460–GQ129463. The Neighbour-joining analyses consisting of 61 different haplotypes representing all available *cox*1 sequences in GenBank, show that three of the sequences from Romania (RO1-3) belong to a separate haplogroup, while all four sequences form a group, although with low support (47 % bootstrap), with a sequence (GQ370816) recovered from rainbow trout in Italy [2] (Fig. 2). The K2-distance between RO1-3 was only 0.001 to 0.003, while the distance between RO1-RO3 and RO4 ranged between 0.009–0.011 (Table 3). The genetic distance between the Romanian haplotypes and the common rainbow trout haplotype that is widespread in Europe (haplotype F and RBT according to [17] and [35], respectively) varied between 0.015–0.019 and the distances between the present haplotypes and haplotype GQ370816 from Italy were between 0.005–0.009 (Table 3).

**Table 3:** Genetic distances (Kimura 2-parameter distance) between haplotypes of *Gyrodactylus salaris* Malmberg, 1957 with the number of nucleotide differences in parentheses

Haplotype	F	A	ITA	RO1	RO2	RO3
4, F^a^						
15, A^a^	0.026 (19)					
30, GQ370816, ITA^b^	0.015 (11)	0.022 (16)				
42, GQ129460, RO1^c^	0.015 (11)	0.019 (14)	0.005 (4)			
43, GQ129461, RO2^c^	0.016 (12)	0.021 (15)	0.007 (5)	0.001 (1)		
44, GQ129462, RO3^c^	0.016 (12)	0.021 (15)	0.007 (5)	0.001 (1)	0.003 (2)	
45, GQ129463, RO4^c^	0.019 (14)	0.026 (19)	0.009 (7)	0.009 (7)	0.011 (8)	0.011 (8)

^a^Haplotype codes F and A refer to codes in Hansen et al. [17]

^b^Haplotype from Italy

^c^Haplotypes from Romania

The RO1 haplotype was the most common, occurring in 23 specimens recovered from six farms and on all three hosts examined. RO2 occurred in three farms on rainbow trout and brook trout and RO3 was restricted to one specimen from rainbow trout. RO4 occurred only on two specimens of brook trout in one farm where this was the only host. Several farms were infected with more than one haplotype (Table 3).

## Discussion

To the best of our knowledge, *Gyrodactylus salaris* is reported from Romania for the first time and this extends the known range of this parasite in Europe. The results from the present study and previous studies [2, 3, 36] show that investigations in new countries and localities often results in the finding of *G. salaris*, and thus it is likely that this parasite has an even wider distribution than presently known. The results also adds further to the fact that *G. salaris* is a very common parasite in European rainbow trout farms and again calls for caution and control of live fish movement between aquaculture facilities as has been pointed out earlier [37].

*Gyrodactylus truttae* was also found for the first time in Romania in the current study and this adds more information on the occurrence of this parasite in Europe. The finding in Romania fits well with earlier knowledge on the distribution of this parasite which is said to occur south of the Baltic (Poland, Denmark, Germany, Czech Republic, Slovakia and the UK), but has not been found further north in Sweden, Finland or Norway [1].

Four mitochondrial haplotypes of *G. salaris* were found in Romania and all of these haplotypes are new to science and do not group with strong support with any previously known haplotypes (Fig. 1), see also [14], although one haplotype show a high similarity to a haplotype from Italy [2]. The finding of new haplotypes on rainbow trout is not surprising as there is increasing evidence for an unknown diversity of *G. salaris* haplotypes in farms with rainbow trout and in natural populations of salmon, and of *G. thymalli* haplotypes on grayling [2, 10, 17–19]. As all of the haplotypes found in Romania are novel, it is impossible to establish the origin of infection. The infections can be introduced directly via import of rainbow trout, via anthropogenic introductions to local water sheds and further to fish farms or they can be found natural in the wild in Romania or indeed a combination of these possibilities. Rainbow trout is an introduced species to Europe (and thus to Romania) and although it is not unlikely that the origin of the infections are via the import of rainbow trout, the haplotypes found cannot be linked to the most common haplotype on rainbow trout in Europe (Haplotype F/RBT) or indeed to any other known haplotypes. The status of *Gyrodactylus* spp. infections on wild salmonids is not known in Romania and thus infections from the natural environment cannot be assessed at the moment. However, based on the fact that the water in the farms is filtered through a sand filter, it is not suspected that the intake water is the source of the infections inside the farm. The presence of the same haplotypes in several farms, however, definitely shows that the parasite is being spread within Romania with movement of infected fish between facilities and points to the fact that rainbow trout is an important host in spreading the parasite within the country and this may also represent the means by which the parasite was introduced into Romania. The highest genetic difference between the haplotypes found in Romania is equivalent to what is seen between the isolate from Atlantic salmon in River Göta (Sweden) and other isolates (e.g. in nearby rivers) and River Göta is considered to have had a separate introduction history [17]. It is likely that the haplotypes found in Romania might have been introduced from several geographically isolated localities. The current study also describes the presence of more than one haplotype of *G. salaris* in farms which might point to repeated introduction/stocking to the farms and maybe from several different localities.

*Gyrodactylus salaris* was found on all three salmonids examined and this is the first time *G. salaris* is found on brook trout except for in experimental conditions [38, 39]. Although *G. salaris* has been shown in a number of earlier studies to be able to infect a number of host species under experimental conditions (see [38]), it is important to note that it was found on brook trout at two farm sites, i.e. Tismana Monastery trout farm and Văliug-Semenic trout farm, where rainbow trout was not present. In at least one of the farms, where the water is considered too cold for rearing rainbow trout and where the farm also keeps their own brood stock of brook trout, no other species than the latter had been reared on site. The finding of *G. salaris* thus indicates that it is able to survive and reproduce also on brook trout for an extended period. Thus, transport and stocking of this host should be performed with caution as it might carry infections of *G. salaris*.

The presence of new haplotypes in Romania which cannot reliably be linked to other haplotypes or haplogroups previously published [14] and are not recovered from salmon or grayling again raises the taxonomic question on whether the specimens should be labelled *G. salaris* or *G. thymalli*. Salmon has never been present in Romania while grayling is present (IUCN red list: http://maps.iucnredlist.org), but not in the area where the current study was carried out. As rainbow trout, from where most specimens of *Gyrodactylus* in the present study were found, is an introduced species to Europe and not the original host of *G. salaris* or *G. thymalli*, host-based identification cannot be used for the Romanian specimens recovered here and should be considered with caution. Morphology and morphometric analyses cannot differentiate between *G. salaris* and *G. thymalli* [8, 16] and thus we are left with the molecules. ITS in all instances cannot help differentiate specimens that are *G. salaris* or *G. thymalli* [10, 17, 40] and when analyses of *cox*1 haplotypes in the present study show that the specimens from Romania are new and not particularly related to any of the other known haplotypes, the taxonomic conclusion is not straightforward. As the name *G. salaris* has been applied for all isolates from other hosts than grayling so far and was the first described of the two *Gyrodactylus* spp., we have nevertheless chosen to use this name for the Romanian isolates.

The present study shows the importance of screening procedures also for diseases and infections that do not cause clinical signs of disease. It would be of importance and interest to carry out screening of wild salmonids in Romania to establish the status of infection with gyrodactylids. This could aid in establishing or ruling out the source of the current infections with *G. salaris* (and other *Gyrodactylus* spp.) in Romanian fish farms. It is also of importance to carry out controlled infections experiments to establish the possible virulence of these new strains towards Atlantic salmon.

As highlighted earlier, it seems more than likely that the examination of farms with *O. mykiss* in other countries will extend the range of *G. salaris* further [2]. This study also demonstrates that brook trout can act as a good host and vector for *G. salaris*.

## Conclusions

*Gyrodactylus salaris* was discovered on farmed salmonids in Romania for the first time. Four new mitochondrial haplotypes that were not identical or phylogenetically connected to any other known haplotypes were found and thus the origin of the infections is unknown. *Gyrodactylus salaris* was found to infect brook trout on a farm where no other salmonid hosts were present or had been reared. The present results adds further proof to the fact that rainbow trout is an important host in the spreading of *G. salaris* between farms and that the transport and stocking of rainbow trout should be done only after careful examination of the fish for the presence of this parasite. The virulence of the variants (haplotypes) found in the present study is unknown and requires establishing.

## Abbreviations

*Cox*1, cytochrome oxidase *c* subunit 1; ITS, internal transcribed spacer; OiE, Organisation Mondiale de la Santé Animale/World Organisation for Animal Health

## Additional files

Additional file 1: Table S1. List of haplotypes included in the 745 bp *cox*1 alignment, their GenBank accession numbers, fish host and country of origin. The haplotypes 40 and 41, representing introgressed sequences (see [37]) were not included in any of the analyses, but are included in this table for information. A number of other haplotypes were left out because they were shorter than 745 bp, but these were not unique haplotypes. (XLSX 21 kb) Additional file 2: Alignment (FASTA-format) (745 bp) of *cox*1 haplotypes used in the present study. (DOCX 22 kb)
