# Supplementary material for: Infections with Gyrodactylus spp. (Monogenea) in Romanian fish farms: Gyrodactylus salaris Malmberg, 1957 extends its range
Source: Parasit Vectors. 2016 Aug 11;9:444. doi: 10.1186/s13071-016-1727-7 (PMC4981988; doi:10.1186/s13071-016-1727-7)
Supplement: Additional file 2: — Alignment (FASTA-format) (745 bp) of cox1 haplotypes used in the present study. (DOCX 22 kb) [file 13071_2016_1727_MOESM2_ESM.docx]

**Additional file 2**

**Alignment of partial (745 bp) mitochondrial *cox*1 gene sequences from *Gyrodactylus salaris* and *Gyrodactylus thymalli* (Monogenea) and outgroup, *G. derjavinoides***

Aligned using MUSCLE as implemented in Mega6.

Sequences numbered 40 and 41 were removed prior to submission as they represent introgressed sequences (see Ziętara et al., 2010. Introgression of distant mitochondria into the genome of *Gyrodactylus salaris*: Nuclear and mitochondrial markers are necessary to identify parasite strains. Acta Parasitologica, 55, 20-28.)

>1

GCTATTGATGCCATCAATGGTGCTAGTATTCGCTAGTATGTGGTTCGGCAGTGGGACAGGATGAACATTTTACCCACCACTGTCCGGGGCCAGGTCTAGTCCAAGCATTGGTACCGACTTTTTGATGTTTTCGCTTCACCTGTCTGGTATTTCCAGTATATTCAGCTCATTAAACTTTATATGTACCATTATAAGAGCTTGAGGTGTGTCCGTGAATATTAAGGACACTGCTATAGTTATATGGGCTTACTTGTTTACGTCCATCTTACTTATACTGTCCCTACCAGTGCTAGCCGCTGGGATAACAATGCTGTTATTTGACCGGAACTTCAACTCATCCTTCTTTGACCCAGTGGGCGGAGGGGACCCAGTCCTATTCCAACACTTATTTTGGTTCTTTGGCCACCCAGAGGTGTACGTACTAATACTCCCAGCATTCGGTATGATTAGTCACATCTGTATAACGTTAAGTAAAGGAGAGCAACCATTTGGTTACTACGGTATGGTGTTCGCCATGTTCTCTATAGTCTGCTTAGGTAGGGTAGTATGAGCCCACCATATGTTCTCTATAGGTATGGATGTAAAGACTTCAGTCTTTTTCAGGTCTGTAACTATGATAATTGCGGTACCCACGGGTATCAAGATCTTCACGTGACTTTACATGCTAACCAGAAGCTCAAAAAAGGCCAATAACCCAATCGTGTGGTGGGTGTACGGCTTCATAATACTGTTTACTATCGGAGGA

>2

GCTATTGATGCCATCAATGGTGCTAGTATTCGCTAGTATGTGGTTCGGCAGTGGGACAGGATGAACATTTTACCCACCACTGTCCGGGGCCAGGTTTAGTCCAAGCATTGGTACCGACTTTTTGATGTTTTCGCTTCACCTGTCTGGTATTTCCAGTATATTCAGCTCATTAAACTTTATATGTACCATTATAAGAGCTTGAGGTGTGTCCGTGAATATTAAGGACACTGCTATAGTTATATGGGCTTACTTGTTTACGTCCATCTTACTTATACTGTCCCTACCAGTGCTAGCCGCTGGGATAACAATGCTGTTATTTGACCGGAACTTCAACCCATCCTTCTTTGACCCAGTGGGCGGAGGGGACCCAGTCCTATTCCAACACTTATTTTGGTTCTTTGGCCACCCAGAGGTGTACGTACTAATACTCCCAGCATTCGGTATGATTAGTCACATCTGTATAACGTTAAGTAAAGGAGAGCAACCATTTGGTTACTACGGTATGGTGTTCGCCATGTTCTCTATAGTCTGCTTAGGTAGGGTAGTATGAGCCCACCATATGTTCTCTATAGGTATGGATGTAAAGACTTCAGTCTTTTTCAGGTCTGTAACTATGATAATTGCGGTACCCACGGGTATCAAGATCTTCACGTGACTTTACATGCTAACCAGAAGCTCAAAAAAGGCCAATAACCCAATCGTGTGGTGGGTGTACGGCTTCATAATACTGTTTACTATCGGAGGA

>3

GCTATTGATGCCATCAATGGTGCTAGTATTCGCTAGTATGTGGTTCGGCAGTGGGACAGGATGAACATTTTACCCACCACTGTCCGGGGCCAGGTTTAGTTCAAGCATTGGTACCGACTTTTTGATGTTTTCGCTTCACCTGTCTGGTATTTCCAGTATATTCAGCTCATTAAACTTTATATGTACCATTATAAGAGCTTGAGGTGTGTCCGTGAATATTAAGGACACTGCTATAGTTATATGGGCTTACTTGTTTACGTCCATCTTACTTATACTGTCCCTACCAGTGCTAGCCGCTGGGATAACAATGCTGTTATTTGACCGGAACTTCAACTCATCCTTCTTTGACCCAGTGGGCGGAGGGGACCCAGTCCTATTCCAACACTTATTTTGGTTCTTTGGCCACCCAGAGGTGTACGTACTAATACTCCCAGCATTCGGTATGATTAGTCACATCTGTATAACGTTAAGTAAAGGAGAGCAACCATTTGGTTACTACGGTATGGTGTTCGCCATGTTCTCTATAGTCTGCTTAGGTAGGGTAGTATGAGCCCACCATATGTTCTCTATAGGTATGGATGTAAAGACTTCAGTCTTTTTCAGGTCTGTAACTATGATAATTGCGGTACCCACGGGTATCAAGATCTTCACGTGACTTTACATGCTAACCAGAAGCTCAAAAAAGGCCAATAACCCAATCGTGTGGTGGGTGTACGGCTTCATAATACTGTTTACTATCGGAGGA

>4

GCTATTGATGCCATCAATGGTGCTAGTATTCGCTAGTATGTGGTTCGGCAGTGGGACAGGATGAACATTTTACCCACCACTGTCCGGGGCCAGGTTTAGTCCAAGCATTGGTACCGACTTTTTGATGTTTTCGCTTCACCTGTCTGGTATTTCCAGTATATTCAGCTCATTAAACTTTATATGTACCATTATAAGAGCTTGAGGTGTGTCCGTGAATATTAAGGACACTGCTATAGTTATATGGGCTTACTTGTTTACGTCCATCTTACTTATACTGTCCCTACCAGTGCTAGCCGCTGGGATAACAATGCTGTTATTTGACCGGAACTTCAACTCATCCTTCTTTGACCCAGTGGGCGGAGGGGACCCAGTCCTATTCCAACACTTATTTTGGTTCTTTGGCCACCCAGAGGTGTACGTACTAATACTCCCAGCATTCGGTATGATTAGTCACATCTGTATAACGTTAAGTAAAGGAGAGCAACCATTTGGTTACTACGGTATGGTGTTCGCCATGTTCTCTATAGTCTGCTTAGGTAGGGTAGTATGAGCCCACCATATGTTCTCTATAGGTATGGATGTAAAGACTTCAGTCTTTTTCAGGTCTGTAACTATGATAATTGCGGTACCCACGGGTATCAAGATCTTCACGTGACTTTACATGCTAACCAGAAGCTCAAAAAAGGCCAATAACCCAATCGTGTGGTGGGTGTACGGCTTCATAATACTGTTTACTATCGGAGGA

>5

GCTATTGATGCCATCAATGGTGCTAGTATTCGCTAGTATGTGGTTCGGCAGTGGGACAGGATGAACATTTTACCCACCACTGTCCGGGGCCAGGTTTAGTCCAAGCATTGGTACCGACTTTTTGATGTTTTCGCTTCACCTGTCTGGTATTTCCAGTATATTCAGCCCATTAAACTTTATATGTACCATTATAAGAGCTTGAGGTGTGTCCGTGAATATTAAGGACACTGCTATAGTTATATGGGCTTACTTGTTTACGTCCATCTTACTTATACTGTCCCTACCAGTGCTAGCCGCTGGGATAACAATGCTGTTATTTGACCGGAACTTCAACTCATCCTTCTTTGACCCAGTGGGCGGAGGGGACCCAGTCCTATTCCAACACTTATTTTGGTTCTTTGGCCACCCAGAGGTGTACGTACTAATACTCCCAGCATTCGGTATGATTAGTCACATCTGTATAACGTTAAGTAAAGGAGAGCAACCATTTGGTTACTACGGTATGGTGTTCGCCATGTTCTCTATAGTCTGCTTAGGTAGGGTAGTATGAGCCCACCATATGTTCTCTATAGGTATGGATGTAAAGACTTCAGTCTTTTTCAGGTCTGTAACTATGATAATTGCGGTACCCACGGGTATCAAGATCTTCACGTGACTTTACATGCTAACCAGAAGCTCAAAAAAGGCCAATAACCCAATCGTGTGGTGGGTGTACGGCTTCATAATACTGTTTACTATCGGAGGA

>6

GCTATTGATGCCATCAATGGTACTAGTATTCGCTAGTATGTGGTTCGGCAGTGGGACAGGATGAACATTTTACCCACCACTGTCCGGGGCCAGGTTTAGTCCAAGCATTGGTACCGACTTTTTGATGTTTTCGCTTCACCTGTCTGGTATTTCTAGTATATTCAGCTCATTAAACTTTATATGTACCATTATAAGAGCTTGAGGTGTGTCCGTGAATATTAAGGATACTGCTATAGTTATATGGGCTTACTTGTTTACGTCCATCTTACTTATATTGTCCCTACCAGTGCTAGCCGCTGGGATAACAATGCTGTTATTTGACCGGAACTTCAACTCATCATTCTTTGACCCAGTGGGCGGAGGAGACCCAGTCCTATTTCAACACTTATTTTGGTTCTTTGGTCACCCAGAGGTGTACGTGTTAATACTCCCGGCATTCGGTATGATTAGTCACATCTGTATAACGTTAAGTAAAGGAGAGCAACCATTTGGTTACTACGGTATGGTGTTCGCCATGTTCTCTATAGTCTGCTTAGGTAGGGTAGTATGAGCCCACCACATGTTCTCTATAGGTATGGATGTAAAGACTTCAGTCTTTTTCAGGTCTGTAACTATGATAATTGCGGTACCCACGGGTATCAAGATCTTCACGTGACTTTACATGCTAACCAGAAGCTCAAAAAAGGCCAATAACCCAATCGTGTGGTGGGTGTACGGCTTCATAATACTGTTTACTATCGGAGGG

>7

GCTATTGATGCCATCAATGGTGCTAGTATTCGCTAGTATGTGGTTCGGCAGTGGGACAGGATGAACATTTTACCCACCACTGTCCGGGGCCAGGTTTAGTCCAAGCATTGGTACCGACTTTTTGATGTTTTCGCTTCACCTGTCTGGTATTTCCAGTATATTCAGCTCATTAAACTTTATATGTACCATTATAAGAGCTTGAGGTGTGTCCGTGAATATTAAGGACACTGCTATAGTTATATGGGCTTACTTGTTTACGTCCATCTTACTTATACTGTCCCTACCAGTGCTAGCCGCTGGGATAACAATGCTGTTATTTGACCGGAACTTCAACTCATCCTTCTTTGACCCAGTGGGCGGAGGGGACCCAGTCCTATTCCAACACTTATTTTGGTTCTTTGGCCACCCAGAGGTGTACGTACTAATACTCCCAGCATTCGGTATGATTAGTCACATCTGTATAACGTTAAGTAAAGGAGAGCAACCATTTGGTTACTACGGTATGGTGTTCGCCATGTTCTCTATAGTCTGCTTAGGTAGGGTAGTATGAGCCCACCATATGTTCTCTATAGGTATGGATGTAAAGACTTCAGTCTTTTTCAGGTCTGTAACTATGATAATTGCGGTACCCACGGGTATCAAGATCTTCACGTGGCTTTACATGCTAACCAGAAGCTCAAAAAAGGCCAATAACCCAATCGTGTGGTGGGTGTACGGCTTCATAATACTGTTTACTATCGGAGGA

>8

GCTATTGATGCCATCAATGGTGCTAGTATTCGCTAGTATGTGGTTCGGCAGTGGGACAGGATGAACATTTTACCCACCACTGTCCGGGGCCAGGTTTAGTCCAAGCATTGGTACCGACTTTTTGATGTTTTCGCTTCACCTGTCTGGTATTTCCAGTATATTCAGCTCATTAAACTTTATATGTACCATTATAAGAGCTTGAGGTGTGTCCGTGAATATTAAGGACACTGCTATAGTTATATGGGCTTACTTGTTTACGTCCATCTTACTTATACTGTCCCTACCAGTGCTAGCCGCTGGGATAACAATGCTGTTATTTGACCGGAACTTCAACTCATCCTTCTTTGACCCAGTGGGCGGAGGGGACCCAGTCCTATTCCAACACTTATTTTGGTTCTTTGGCCGCCCAGAGGTGTACGTACTAATACTCCCAGCATTCGGTATGATTAGTCACATCTGTATAACGTTAAGTAAAGGAGAGCAACCATTTGGTTACTACGGTATGGTGTTCGCCATGTTCTCTATAGTCTGCTTAGGTAGGGTAGTATGAGCCCACCATATGTTCTCTATAGGTATGGATGTAAAGACTTCAGTCTTTTTCAGGTCTGTAACTATGATAATTGCGGTACCCACGGGTATCAAGATCTTCACGTGACTTTACATGCTAACCAGAAGCTCAAAAAAGGCCAATAACCCGATCGTGTGGTGGGTGTACGGCTTCATAATACTGTTTACTATCGGAGGA

>9

GCTATTGATGCCATCAATGGTGCTAGTATTCGCTAGTGTGTGGTTCGGTAGTGGGACAGGATGAACATTTTACCCACCACTGTCCGGGGCCAGGTTTAGTCCAAGCATTGGTACCGACTTTTTGATGTTTTCGCTTCACCTGTCTGGTATTTCCAGTATATTCAGCTCATTAAACTTTATATGTACCATTATAAGAGCTTGAGGTGTGTCCGTGAATATTAAGGACACTGCTATAGTTATATGGGCTTACTTGCTTACGTCCATCTTACTTATACTGTCCCTACCAGTGCTAGCCGCTGGGATAACAATGCTGTTATTTGACCGGAACTTCAACTCATCCTTCTTTGACCCAGTGGGCGGAGGGGACCCAGTCCTATTCCAACACTTATTTTGGTTCTTTGGCCACCCAGAGGTGTACGTACTAATACTCCCAGCATTCGGTATGATTAGTCACATCTGTATAACGTTAAGTAAAGGAGAGCAACCATTTGGTTACTACGGTATGGTGTTCGCCATGTTCTCTATAGTCTGCTTAGGTAGGGTAGTATGAGCCCACCATATGTTCTCTATAGGTATGGATGTAAAGACTTCAGTCTTTTTCAGGTCTGTAACTATGATAATTGCGGTACCCACGGGTATCAAGATCTTCACGTGACTTTACATGCTAACCAGAAGCTCAAAAAAGGCCAATAACCCAATCGTGTGGTGGGTGTACGGCTTCATAATACTGTTTACTATCGGAGGA

>10

GCTATTGATGCCATCAATGGTGCTAGTATTCGCTAGTATGTGGTTCGGCAGTGGGACAGGATGAACATTTTACCCACCACTGTCCGGGGCCAGGTTTAGTCCAAGCATTGGTACCGACTTTTTGATGTTTTCGCTTCACCTGTCTGGTATTTCTAGTATATTCAGCTCATTAAACTTTATATGTACCATTATAAGAGCTTGAGGTGTGTCCGTGAATATTAAGGATACTGCTATAGTTATGTGGGCTTACTTGTTTACGTCCATCTTACTTATATTGTCCCTACCAGTGCTAGCCGCTGGGATAACAATGCTGTTATTTGACCGGAACTTCAACTCATCATTCTTTGACCCAGTGGGCGGAGGAGACCCAGTCCTATTTCAACACTTATTTTGGTTCTTTGGTCACCCAGAGGTGTACGTGTTAATACTCCCGGCATTCGGTATGATTAGCCACATCTGTATAACGTTAAGTAAAGGAGAGCAACCATTTGGTTACTACGGTATGGTGTTCGCCATGTTCTCTATAGTCTGCTTAGGTAGGGTAGTATGAGCCCACCACATGTTCTCTATAGGTATGGATGTAAAGACTTCAGTCTTTTTCAGGTCTGTAACTATGATAATTGCGGTACCCACGGGTATCAAGATCTTCACGTGACTTTACATGCTAACCAGAAGCTCAAAAAAGGCCAATAACCCAATCGTGTGGTGGGTGTACGGCTTCATAATACTGTTTACTATCGGAGGG

>11

GCTATTGATGCCATCAATGGTGCTAGTATTCGCTAGTATGTGGTTCGGCAGTGAGACAGGATGAACATTTTACCCACCACTGTCCGGGGCCAGGTTTAGTCCAAGCATTGGTACCGACTTTTTGATGTTTTCGCTTCACCTGTCTGGTATTTCCAGTATACTCAGCTCATTAAACTTTATATGTACCATTATAAGAGCTTGAGGTGTGTCCGTGAATATTAAGGACACTGCTATAGTTATATGGGCTTACTTGTTTACGTCCATCTTACTTATACTGTCCCTACCAGTGCTAGCCGCTGGGATAACAATGCTGTTATTTGACCGGAACTTCAACTCATCCTTCTTTGACCCAGTGGGCGGAGGGGACCCAGTCCTATTCCAACACTTATTTTGGTTCTTTGGCCACCCAGAGGTGTACGTACTAATACTCCCAGCATTCGGTATGATTAGTCACATCTGTATAACGTTAAGTAAAGGAGAGCAACCATTTGGTTACTACGGTATGGTGTTCGCCATGTTCTCTATAGTCTGCTTAGGTAGGGTAGTATGAGCCCACCATATGTTCTCTATAGGTATGGTTGTAAAGACTTCAGTCTTTTTCAGGTCTGTAACTATGATAATTGCGGTACCCACGGGTATCAAGATCTTCACGTGACTTTACATGCTAACCAGAAGCTCAAAAAAGGCCAATAACCCAATCGTGTGGTGGGTGTACGGCTTCATAATACTGTTTACTATCGGAGGA

>12

GCTATTGATGCCATCAATGGTGTTAGTATTCGCCAGTATGTGGTTCGGCAGTGGAACAGGATGAACATTTTACCCACCACTGTCCGGGGCCAGGTTTAGTCCAAGCATTGGTACCGACTTTTTGATGTTTTCGCTTCACCTGTCTGGTATCTCTAGCATATTCAGCTCATTAAACTTTATATGCACCATTATAAGAGCTTGAGGTGTGTCCGTGAATATTAAGGACACTGCTATAGTTATATGGGCTTACTTGTTTACATCCATCTTACTTATATTGTCTCTACCAGTGCTAGCCGCTGGGATAACAATGCTGTTATTTGACCGGAACTTCAACTCATCATTCTTTGACCCAGTGGGTGGAGGGGACCCAGTCCTATTTCAACACTTATTTTGGTTCTTTGGCCACCCAGAGGTGTACGTGCTAATACTCCCGGCATTCGGTATGATTAGTCACATTTGTATAACGTTGAGTAAAGGAGAGCAACCATTTGGTTACTACGGCATGGTGTTCGCCATGTTCTCTATAGTCTGTTTAGGTAGGGTAGTATGAGCCCACCATATGTTCTCTATAGGTATGGATGTAAAGACTTCAGTCTTTTTCAGGTCTGTAACTATGATAATTGCGGTACCCACGGGTATCAAGATCTTCACGTGACTTTACATGCTAACCAGAAGCTCAAAAAAGGCCAATAACCCAATCGTGTGGTGGGTGTACGGCTTCATAATACTGTTTACTATCGGAGGG

>13

GCTATTGATGCCATCAATGGTGTTAGTATTCGCCAGTATGTGGTTCGGCAGTGGAACAGGATGAACATTTTACCCACCACTGTCCGGGGCCAGGTTTAGTCCAAGCATTGGTACCGACTTTTTGATGTTTTCGCTTCACCTGTCTGGTATATCTAGTATATTCAGCTCATTAAACTTTATATGCACCATTATAAGAGCTTGAGGTGTGTCCGTGAATATTAAGGACACTGCTATAGTTATATGGGCTTACTTGTTTACATCCATCTTACTTATATTGTCCCTACCAGTGCTAGCCGCTGGGATAACAATGCTGTTATTTGACCGGAACTTCAACTCATCATTCTTTGACCCAGTGGGCGGAGGGGACCCAGTCCTATTTCAACACTTATTTTGGTTCTTTGGCCACCCAGAGGTGTACGTGCTAATACTCCCGGCATTCGGTATGATTAGTCACATTTGTATAACGTTGAGTAAAGGAGAGCAACCATTTGGTTACTACGGCATGGTGTTCGCCATGTTCTCTATAGTCTGTTTAGGTAGGGTAGTATGAGCCCACCATATGTTCTCTATAGGTATGGATGTAAAGACTTCAGTCTTTTTCAGGTCTGTAACTATGATAATTGCGGTACCCACGGGTATCAAGATCTTCACGTGACTTTACATGCTAACCAGAAGCTCAAAAAAGGCCAATAACCCAATCGTGTGGTGGGTGTACGGCTTCATAATACTGTTTACTATCGGAGGG

>14

GCTATTGATGCCATCAATGGTGTTAGTATTCGCCAGTGTGTGGTTCGGCAGTGGAACAGGATGAACATTTTACCCACCACTGTCCGGGGCCAGGTTTAGTCCAAGCATTGGTACCGACTTTTTGATGTTTTCGCTTCACCTGTCTGGTATCTCTAGTATATTCAGCTCATTAAACTTTATATGCACCATTATAAGAGCTTGAGGTGTGTCCGTGAGTATTAAGGACACTGCTATAGTTATATGGGCTTACTTGTTTACATCCATCTTACTTATATTGTCCCTACCAGTGCTAGCCGCTGGGATAACAATGCTGTTATTTGACCGGAACTTCAACTCATCATTCTTTGACCCAGTGGGCGGAGGGGACCCAGTCCTATTTCAACACTTATTTTGGTTCTTTGGCCACCCAGAGGTGTACGTGCTAATACTCCCGGCATTCGGTATGATTAGTCACATTTGTATAACGTTGAGTAAAGGAGAGCAACCATTTGGTTACTACGGCATGGTGTTCGCCATGTTCTCTATAGTCTGTTTAGGTAGGGTAGTATGAGCCCACCATATGTTCTCTATAGGTATGGATGTAAAGACTTCAGTCTTTTTCAGGTCTGTAACTATGATAATTGCGGTACCCACGGGTATCAAGATCTTCACGTGACTTTACATGCTAACCAGAAGCTCAAAAAAGGCCAATAACCCAATCGTGTGGTGGGTGTACGGCTTCATAATACTGTTTACTATCGGAGGG

>15

GCTATTGATGCCATCAATGGTGTTAGTATTCGCCAGTATGTGGTTCGGCAGTGGAACAGGATGAACATTTTACCCACCACTGTCCGGGGCCAGGTTTAGTCCAAGCATTGGTACCGACTTTTTGATGTTTTCGCTTCACCTGTCTGGTATCTCTAGTATATTCAGCTCATTAAACTTTATATGCACCATTATAAGAGCTTGAGGTGTGTCCGTGAATATTAAGGACACTGCTATAGTTATATGGGCTTACTTGTTTACATCCATCTTACTTATATTGTCCCTACCAGTGCTAGCCGCTGGTATAACAATGCTGTTATTTGACCGGAACTTCAACTCATCATTCTTTGACCCAGTGGGCGGAGGGGACCCAGTCCTATTTCAACACTTATTTTGGTTCTTTGGCCACCCAGAGGTGTACGTGCTAATACTCCCGGCATTCGGTATGATTAGTCACATTTGTATAGCGTTGAGTAAAGGAGAGCAACCATTTGGTTACTACGGCATGGTGTTCGCCATGTTCTCTATAGTCTGTTTAGGTAGGGTAGTATGAGCCCACCATATGTTCTCTATAGGTATGGATGTAAAGACTTCAGTCTTTTTCAGGTCTGTAACTATGATAATTGCGGTACCCACGGGTATCAAGATCTTCACGTGACTTTACATGCTAACCAGAAGCTCAAAAAAGGCCAATAACCCAATCGTGTGGTGGGTGTACGGCTTCATAATACTGTTTACTATCGGAGGG

>16

GCTATTGATGCCATCAATGGTGCTAGTATTCGCTAGTATGTGGTTCGGCAGTGGAACAGGATGAACATTTTACCCACCACTGTCCGGGGCCAGGTTTAGTCCAAGCATTGGTACCGACTTTTTGATGTTTTCGCTTCACCTGTCTGGTATTTCTAGTATATTCAGCTCATTAAACTTCATATGCACCATTATAAGAGCTTGAGGTGTGTCCGTGAATATTAAGGACACTGCTATAGTTATATGGGCTTACTTGTTTACGTCCATCTTACTTATATTGTCCCTACCAGTGCTAGCCGCTGGGATAACAATGCTGTTATTTGACCGAAACTTCAACTCATCCTTCTTTGACCCAGTGGGCGGAGGGGACCCAGTCCTATTTCAACACTTATTTTGGTTCTTTGGTCACCCAGAGGTTTACGTGCTAATACTCCCGGCATTCGGTATGATTAGTCACATCTGTATAACGTTAAGTAAAGGAGAGCAACCATTTGGTTACTACGGTATGGTGTTCGCCATGTTCTCTATAGTCTGTTTAGGTAGGGTAGTGTGAGCCCACCACATGTTCTCTATAGGTATGGATGTAAAGACTTCAGTCTTTTTCAGGTCTGTAACTATGATAATTGCGGTACCCACGGGCATCAAGATCTTCACGTGACTTTACATGCTAACCAGAAGCTCAAAAAAGGCCAATAACCCAATCGTGTGGTGGGTGTACGGCTTCATAATACTGTTTACTATCGGAGGA

>17

GCTATTGATGCCATCAATGGTGCTAGTATTCGCTAGTATGTGGTTCGGCAGTGGAACAGGATGAACATTTTACCCACCACTGTCCGGGGCCAGGTTTAGTCCAAGCATTGGTACCGACTTTTTGATGTTTTCGCTTCACCTGTCTGGTATCTCTAGTATATTGAGCTCATTAAACTTTATATGCACCATTATAAGAGCTTGAGGTGTGTCCGTGAATATTAAGGACACTGCTATAGTTATATGGGCTTACTTGTTTACGTCCATCTTGCTTATATTGTCCCTACCAGTGCTAGCCGCTGGGATAACAATGCTGTTATTTGACCGGAACTTCAACTCATCATTCTTTGACCCAGTGGGCGGAGGGGACCCAGTCCTATTTCAACACTTATTTTGGTTCTTTGGTCACCCAGAGGTGTACGTGCTAATACTCCCGGCATTCGGTATGATTAGTCACATCTGTATAACGCTAAGTAACGGAGAGCAACCATTTGGTTACTACGGCATGGTGTTCGCCATGTTCTCTATAGTCTGTTTAGGTAGGGTAGTATGGGCCCACCATATGTTCTCTATAGGTATGGATGTAAAGACTTCAGTCTTTTTCAGGTCTGTAACTATGATAATTGCGGTACCCACGGGTATCAAGATCTTTACGTGACTTTACATGCTAACCAGAAGCTCAAAAAAGGCCAATAACCCAATCGTGTGGTGGGTGTACGGCTTCATAATACTGTTTACTATCGGAGGG

>18

GCTATTGATGCCATCAATGGTGTTAGTATTCGCCAGTATGTGGTTCGGCAGTGGAACAGGATGAACATTTTACCCACCACTGTCCGGGGCCAGGTTTAGTCCAAGCATTGGTACCGACTTTTTGATGTTTTCGCTTCACCTGTCTGGTATCTCTAGTATATTCAGCTCATTAAACTTTATATGCACCATTATAAGAGCTTGAGGTGTGTCCGTGAATATTAAGGACACTGCTATAGTTATATGGGCTTACTTGTTTACATCCATCTTACTTATATTGTCCCTACCAGTGCTAGCCGCTGGGATAACAATGCTGTTATTTGACCGGAACTTCAACTCATCATTCTTTGACCCAGTGGGCGGAGGGGACCCAGTCCTATTTCAACACTTATTTTGGTTCTTTGGCCACCCAGAGGTGTACGTGCTAATACTCCCGGCATTCGGTATGATTAGTCACATTTGTATAACGTTGAGTAAAGGAGAGCAACCATTTGGTTACTACGGCATGGTGTTCGCCATGTTCTCTATAGTCTGTTTAGGTAGGGTAGTATGAGCCCACCATATGTTCTCTATAGGTATGGATGTAAAGACTTCAGTCTTTTTCAGGTCTGTAACTATGATAATTGCGGTACCCACGGGTATCAAGATCTTCACGTGACTTTACATGCTAACCAGAAGCTCAAAAAAGGCCAATAACCCAATCGTGTGGTGGGTGTACGGCTTCATAATACTGTTTACTATCGGAGGG

>19

GCTATTGATGCCATCAATGGTGCTAGTATTCGCTAGTATATGGTTCGGCAGTGGAACAGGATGAACATTTTACCCACCMCTGTCCGGGGCCAGGTTTAGWCCAAGYATYGGTACCGACTTTTTGATGTTWTCGCTYCACCTGTCKGGTATTTCTAGTATATTCAGCTCATTAAACTTTATATGCACCATTATAAGAGCTTGAGGTGTGTCCGTGAATATTAAGGACACTGCTATAGTTATATGGGCTTACTTGTTTACGTCCATCTTACTTATATTGTCCCTACCAGTGCTAGCCGCTGGGATAACAATGCTGTTATTTGACCGGAACTTCAACTCATCATTCTTTGACCCAGTGGGCGGAGGGGACCCAGTCCTATTTCAACACTTATTTTGGTTCTTTGGTCACCCAGAGGTGTACGTGCTAATACTCCCGGCATTCGGTATGATTAGTCACATCTGTATAACGYTAAGTAAAGGAGAGCAACCATTTGGTTACTACGGTATGGTGTTCGCCATGTTCTCTATAGTCTGTTTAGGTAGGGTAGTATGAGCCCACCATATGTTCTCTATAGGTATGGATGTAAAGACTTCAGTCTTTTTCAGGTCTGTAACTATGGTAATTGCGGTACCCACGGGTATCAAGATTTTCACGTGACTTTACATGCTAGCCAGAAGCTCAAAAAAGGCCAATAACCCAATCGTGTGGTGGGTGTACGGCTTCATAATACTGTTTACTATCGGAGGA

>20

GCTATTGATGCCATCAATGGTGCTAGTATTCGCTAGTATGTGGTTCGGCAGTGGGACAGGATGAACATTTTACCCACCACTGTCCGGGGCCAGGTTTAGTCCAAGCATTGGTACCGACTTTTTGATGTTCTCGCTTCACCTGTCTGGTATTTCCAGTATATTCAGCTCATTAAACTTTATATGTACCATTATAAGAGCTTGAGGTGTGTCCGTTAATATTAAGGACACTGCTATAGTTATATGGGCTTACTTGTTTACGTCCATCTTACTTATACTGTCCCTACCAGTGCTAGCCGCTGGGATAACAATGCTGTTATTTGACCGGAACTTCAACTCATCCTTCTTTGACCCAGTGGGCGGAGGGGACCCAGTCCTATTCCAACACTTATTTTGGTTCTTTGGCCACCCAGAGGTGTACGTACTAATACTCCCAGCATTCGGTATGATTAGTCACATCTGTATAACGTTAAGTAAAGGAGAGCAACCATTTGGTTACTACGGTATGGTGTTCGCCATGTTCTCTATAGTCTGCTTAGGTAGGGTAGTATGAGCCCACCACATGTTCTCTATAGGTATGGATGTAAAGACTTCAGTCTTTTTCAGGTCTGTAACTATGATAATTGCGGTACCCACGGGTATCAAGATCTTCACGTGACTTTACATGCTAACCAGAAGCTCAAAAAAGGCCAATAACCCAATCGTGTGGTGGGTGTACGGCTTCATAATACTGTTTACTATCGGAGGA

>21

GCTATTAATGCCATCAATGGTGCTAGTATTCGCTAGTATATGGTTCGGCAGTGGAACAGGATGAACATTTTACCCACCACTGTCCGGGGCCAGGTTTAGACCAAGTATCGGTACCGACTTTTTGATGTTTTCGCTTCACCTGTCGGGTATTTCTAGTATATTCAGCTCATTAAACTTTATATGTACCATTATAAGAGCTTGAGGTGTGTCCGTGAATATTAAGGACACTGCTATAGTTATATGGGCTTACTTGTTTACGTCCATCTTACTTATATTGTCCCTACCAGTGCTAGCCGCTGGGATAACAATGCTGTTATTTGACCGGAACTTCAACTCATCATTCTTTGACCCAGTSGGCGGAGGGGACCCAGTCCTATTTCAACACTTATTTTGGTTCTTTGGTCACCCAGAGGTGTACGTGCTAATACTCCCGGCATTCGGTATGATTAGTCATATCTGTATAACGCTAAGTAAAGGAGAGCAACCATTTGGTTACTACGGTATGGTGTTCGCCATGTTCTCTATAGTCTGTTTAGGTAGGGTAGTATGAGCCCACCATATGTTCTCTATAGGTATGGATGTAAAGACTTCAGTCTTTTTCAGGTCTGTAACTATGGTAATTGCGGTACCCACGGGTATCAAGATCTTCACGTGACTTTACATGCTAGCCAGAAGCTCAAAAAAGGCCAATAACCCAATCGTGTGGTGGGTGTACGGCTTCATAATACTGTTTACTATCGGAGGA

>22

GCTATTGATGCCATCAATGGTGCTAGTATTCGCTAGTATGTGGTTCGGCAGTGGAACAGGATGAACATTTTACCCACCACTGTCCGGGGCCAGGTTTAGTCCAAGCATTGGTACCGACTTTTTGATGTTTTCGCTTCACCTGTCTGGTATTTCTAGTATATTCAGCTCATTAAACTTCATATGCACCATTATAAGAGCTTGAGGTGTGTCCGTGAATATTAAGGACACTGCTATAGTTATATGGGCTTACTTGTTTACGTCCATCTTACTTATATTGTCCCTACCAGTGCTAGCCGCTGGGATAACAATGCTGTTATTTGACCGAAACTTCAACTCATCCTTCTTTGACCCAGTGGGCGGAGGGGACCCAGTCCTATTTCAACACTTATTTTGGTTCTTTGGTCACCCAGAGGTTTACGTGCTAATACTCCCGGCATTCGGTATGATTAGTCACATCTGTATAACGTTAAGTAAAGGAGAGCAACCATTTGGTTACTACGGTATGGTGTTCGCCATGTTCTCTATAGTCTGTTTAGGTAGGGTAGTATGGGCCCACCACATGTTCTCTATAGGTATGGATGTAAAGACTTCAGTCTTTTTCAGGTCTGTAACTATGATAATTGCGGTACCCACGGGCATCAAGATCTTCACGTGACTTTACATGCTAACCAGAAGCTCAAAAAAGGCCAATAACCCAATCGTGTGGTGGGTGTACGGCTTCATAATACTGTTTACTATCGGAGGA

>23

GCTATTGATGCCATCAATGGTGCTAGTATTCGCCAGTATGTGGTTCGGCAGTGGAACAGGATGAACATTTTACCCACCACTGTCCGGGGCCAGGTTTAGTCCAAGCATTGGTACCGACTTTTTGATGTTTTCGCTTCACCTGTCTGGTATTTCTAGTATATTCAGCTCATTAAACTTTATATGCACCATTATAAGAGCTTGAGGTGTGTCCGTGAATATTAAGGACACTGCTATAGTTATATGGGCTTACTTGTTTACGTCCATCTTACTTATATTGTCCCTACCAGTGCTAGCCGCTGGGATAACAATGCTGTTATTTGACCGGAACTTCAACTCATCATTCTTTGACCCAGTGGGCGGAGGGGACCCAGTCCTATTTCAACACTTATTTTGGTTCTTTGGCCACCCAGAGGTGTACGTGCTAATACTCCCGGCATTCGGTATGATTAGTCACATCTGTATAACGTTGAGTAAAGGAGAGCAACCATTTGGTTACTACGGCATGGTGTTCGCCATGTTCTCTATAGTCTGTTTAGGTAGGGTAGTATGAGCCCACCACATGTTCTCTATAGGTATGGATGTAAAGACTTCAGTCTTTTTCAGGTCTGTAACTATGATAATTGCGGTACCCACGGGTATCAAGATCTTCACGTGACTTTACATGCTAACCAGAAGCTCAAAAAAGGCCAATAACCCAATCGTGTGGTGGGTGTATGGCTTCATAATACTGTTTACTATCGGAGGG

>24

GCTATTGATGCCATCAATGGTGTTAGTATTTGCCAGTATGTGGTTCGGCAGTGGAACAGGATGAACATTTTACCCACCACTGTCCGGGGCCAGGTTTAGTCCAAGCATTGGTACCGACTTTTTGATGTTTTCGCTTCACCTGTCTGGTATCTCTAGTATATTCAGCTCATTAAACTTTATATGCACCATTATAAGAGCTTGAGGTGTGTCCGTGAATATTAAGGACACTGCTATAGTTATATGGGCTTACTTGTTTACATCCATCTTACTTATATTGTCCCTACCAGTGCTAGCCGCTGGGATAACAATGCTGTTATTTGACCGGAACTTCAACTCATCATTCTTTGACCCAGTGGGCGGAGGGGACCCAGTCCTATTTCAACACTTATTTTGGTTCTTTGGCCACCCAGAGGTGTACGTGCTAATACTCCCGGCATTCGGTATGATTAGTCACATTTGTATAACGTTGAGCAAAGGAGAGCAACCATTTGGTTACTACGGCATGGTGTTCGCCATGTTTTCTATAGTCTGTTTAGGTAGGGTAGTATGAGCCCACCATATGTTCTCTATAGGTATGGATGTAAAGACTTCAGTCTTTTTCAGGTCTGTAACTATGATAATTGCGGTACCCACGGGTATCAAGATCTTCACGTGACTTTACATGCTAACCAGAAGCTCAAAAAAGGCCAATAACCCAATCGTGTGGTGGGTGTACGGCTTCATAATACTGTTTACTATCGGAGGG

>25

GCTATTGATGCCATCAATGGTGCTAGTATTCGCTAGTATATGGTTCGGCAGTGGAACAGGATGAACATTTTACCCACCACTGTCCGGGGCCAGGTTTAGTCCAAGCATTGGTACCGACTTTTTGATGTTTTCGCTTCACCTGTCTGGTATTTCTAGTATATTCAGCTCATTAAACTTTATATGTACCATTATAAGAGCTTGAGGTGTGTCCGTGAATATTAAGGACACTGCTATAGTTATATGGTCTTACTTGTTTACGTCCATCTTACTTATATTGTCCCTACCAGTGCTAGCCGCTGGGATAACAATGCTGTTATTTGATCGGAACTTCAATTCATCATTCTTTGACCCAGTGGGCGGAGGGGACCCAGTCCTATTTCAACACTTATTTTGGTTCTTTGGTCACCCAGAGGTGTACGTGCTAATACTCCCGGCATTCGGCATGATTAGTCACATCTGTATAACGTTAAGTAAAGGAGAGCAACCATTTGGTTACTACGGTATGGTGTTCGCCATGTTCTCTATAGTCTGTTTAGGTAGGGTAGTATGAGCCCACCATATGTTCTCTATAGGCATGGATGTAAAGACTTCAGTCTTTTTCAGGTCTGTAACTATGATAATTGCGGTACCCACGGGTATCAAGATCTTCACGTGACTTTACATGCTAGCCAGAAGCTCAAAAAAGGTCAATAACCCAATCGTGTGGTGGGTGTACGGCTTCATAATACTGTTTACTATCGGAGGA

>26

GCTATTGATGCCATCAATGGTGCTAGTATTCGCTAGTATATGGTTCGGCAGTGGAACAGGATGAACATTTTACCCACCACTGTCCGGGGCCAGGTTTAGACCAAGTATCGGTACCGACTTTTTGATGTTTTCGCTCCACCTGTCGGGTATTTCTAGTATATTCAGCTCATTAAACTTTATATGCACCATTATAAGAGCTTGAGGTGTGTCCGTGAATATTAAGGACACTGCTATAGTTATATGGGCTTACTTGTTTACGTCCATCTTGCTTATATTGTCCCTACCAGTGCTAGCCGCTGGGATAACAATGCTGTTATTTGACCGGAACTTCAACTCATCATTCTTTGACCCAGTGGGCGGAGGGGACCCAGTCCTATTTCAACACTTATTTTGGTTCTTTGGTCACCCAGAGGTGTACGTGCTAATACTCCCGGCATTCGGTATGATTAGTCACATCTGTATAACGCTAAGTAAAGGAGAGCAACCATTTGGTTACTACGGTATGGTGTTCGCCATGTTCTCTATAGTCTGTTTAGGTAGGGTAGTATGAGCCCACCATATGTTCTCTATAGGTATGGATGTAAAGACTTCAGTCTTTTTCAGGTCTGTAACTATGGTAATTGCGGTACCCACGGGTATCAAGATTTTCACGTGACTTTACATGCTAGCCAGAAGCTCAAAAAAGGCCAATAACCCAATCGTGTGGTGGGTGTACGGCTTCATAATACTGTTTACTATCGGAGGA

>27

GCTATTGATGCCATCAATGGTGCTAGTATTCGCCAGTATGTGGTTCGGCAGTGGAACAGGATGAACATTTTACCCACCACTGTCCGGGGCCAGGTTTAGTCCAAGCATTGGTACCGACTTTTTGATGTTTTCGCTTCACCTGTCTGGTATCTCTAGTATATTCAGCTCATTAAACTTTATATGCACCATTATAAGAGCTTGAGGTGTGTCCGTGAATATTAAGGACACTGCTATAGTTATATGGGCTTACTTGTTTACATCCATCTTACTTATACTGTCCCTACCAGTGCTAGCCGCTGGGATAACAATGCTGTTATTTGACCGGAACTTCAACTCATCATTCTTTGACCCAGTGGGCGGAGGGGACCCAGTCCTATTTCAACACTTATTTTGGTTCTTTGGCCACCCAGAGGTGTACGTGCTAATACTCCCGGCATTCGGTATGATTAGTCACATCTGTATAACGTTGAGTAAAGGAGAGCAACCATTTGGTTACTACGGCATGGTGTTCGCCATGTTCTCTATAGTCTGTTTAGGTAGGGTAGTATGAGCCCACCATATGTTCTCTATAGGTATGGATGTAAAGACTTCAGTCTTTTTCAGGTCTGTAACTATGATAATTGCGGTACCCACGGGTATCAAGATCTTCACGTGACTTTACATGCTAACCAGAAGCTCAAAAAAGGCCAATAACCCAATCGTGTGGTGGGTGTACGGCTTCATAATACTGTTTACTATCGGAGGG

>28

GCTATTGATGCCATCAATGGTGCTAGTATTCGCTAGTATATGGTTCGGCAGTGGAACAGGATGAACATTTTACCCACCACTGTCCGGGGCCAGGTTTAGACCAAGTATCGGTACCGACTTTTTGATGTTTTCGCTTCACCTGTCGGGTATTTCTAGTATATTCAGCTCATTAAACTTTATATGCACCATTATAAGAGCTTGAGGTGTGTCCGTGAATATTAAGGACACTGCTATAGTTATATGGGCTTACTTGTTTACGTCCATCTTACTTATATTGTCCCTACCAGTGCTAGCCGCTGGGATAACAATGCTGTTATTTGACCGGAACTTCAACTCATCATTCTTTGACCCAGTGGGCGGAGGGGACCCAGTCCTATTTCAACACTTATTTTGGTTCTTTGGTCACCCAGAGGTGTACGTGCTAATACTCCCGGCATTCGGTATGATTAGTCACATCTGTATAACGCTAAGTAAAGGAGAGCAACCATTTGGTTACTACGGTATGGTGTTCGCCATGTTCTCTATAGTCTGTTTAGGTAGGGTAGTATGAGCCCACCATATGTTCTCCATAGGTATGGATGTAAAGACTTCAGTCTTTTTCAGGTCTGTAACTATGGTAATTGCGGTACCCACGGGTATCAAGATCTTCACGTGACTTTACATGCTAGCCAGAAGCTCAAAAAAGGCCAATAACCCAATCGTGTGGTGGGTGTACGGCTTTATAATACTGTTTACTATCGGAGGA

>29

GCTATTGATGCCATCAATGGTGTTAGTATTCGCCAGTATGTGGTTCGGCAGTGGAACAGGATGAACATTTTACCCACCACTGTCCGGGGCCAGGTTTAGTCCAAGCATTGGTACCGACTTTTTGATGTTTTCGCTTCACCTGTCTGGTATCTCTAGTATATTCAGCTCATTAAACTTTATATGCACCATTATAAGAGCTTGAGGTGTGTCCGTGAATATTAAGGACACTGCTATAGTTATATGGGCTTACTTGTTTACATCCATCTTACTTATATTGTCCCTACCAGTGCTAGCCGCTGGGATAACAATGCTGTTATTTGACCGGAACTTCAACTCATCATTCTTTGACCCAGTGGGCGGAGGGGACCCAGTCCTATTTCAACACTTATTTTGGTTCTTTGGCCACCCAGAGGTGTACGTGCTAATACTCCCGGCATTCGGTATGATTAGTCACATTTGTATAACGTTGAGCAAAGGAGAGCAACCATTTGGTTACTACGGCATGGTGTTCGCCATGTTTTCTATAGTCTGTTTAGGTAGGGTAGTATGAGCCCACCATATGTTCTCTATAGGTATGGATGTAAAGACTTCAGTCTTTTTCAGGTCTGTAACTATGATAATTGCGGTACCCACGGGTATCAAGATCTTCACGTGACTTTACATGCTAACCAGAAGCTCAAAAAAGGCCAATAACCCAATCGTGTGGTGGGTGTACGGCTTCATAATACTGTTTACTATCGGAGGG

>30

GCTATTGATGTCATCAATGGTGCTAGTATTCGCTAGTATATGGTTCGGCAGTGGGACAGGATGAACATTTTACCCACCACTGTCCGGGGCCAGGTTTAGTCCAAGCATTGGTACCGACTTTTTGATGTTTTCGCTTCACCTGTCTGGTATTTCTAGTATATTCAGCTCATTAAACTTTATATGCACCATTATAAGAGCTTGAGGTGTGTCCGTGAATATTAAGGACACTGCTATAGTTATATGGGCTTACTTGTTTACGTCCATCTTACTTATATTGTCCCTACCAGTGCTAGCCGCTGGGATAACAATGCTGTTATTTGACCGGAACTTCAACTCATCATTCTTTGACCCAGTGGGCGGAGGGAACCCAGTCCTATTTCAACACTTATTTTGGTTCTTTGGTCACCCAGAGGTGTACGTGCTAATACTCCCGGCATTCGGTATGATTAGTCACATCTGTATAACGTTAAGTAAAGGAGAGCAACCATTTGGTTACTACGGTATGGTGTTCGCCATGTTCTCTATAGTCTGCTTAGGTAGGGTAGTATGAGCCCACCATATGTTCTCTATAGGTATGGATGTAAAGACTTCAGTCTTTTTCAGGTCTGTAACTATGATAATTGCGGTACCCACGGGTATCAAGATCTTCACGTGACTTTACATGCTAACCAGAAGCTCAAAAAAGGCCAATAACCCAATCGTGTGGTGGGTGTACGGCTTCATAATACTGTTTACTATCGGAGGA

>31

GCTATTGATGCCATCAATGGTGTTAGTATTCGCCAGTGTGTGGTTCGGCAGTGGAACAGGATGAACATTTTACCCACCACTGTCCGGGGCCAGGTTTAGTCCAAGCATTGGTACCGACTTTTTGATGTTTTCGCTTCACCTGTCTGGTATCTCTAGTATATTCAGCTCATTAAACTTTATATGCACCATTATAAGAGCTTGAGGTGTGTCCGTGAGTATTAAGGACACTGCTATAGTTATATGGGCTTACTTGTTTACATCCATCTTACTTATATTGTCCCTACCAGTGCTAGCCGCTGGGATAACAATGCTGCTATTTGACCGGAACTTCAACTCATCATTCTTTGACCCAGTGGGCGGAGGGGACCCAGTCCTATTTCAACACTTATTTTGGTTCTTTGGCCACCCAGAGGTGTACGTGCTAATACTCCCGGCATTCGGTATGATTAGTCACATTTGTATAACGTTGAGTAAAGGAGAGCAACCATTTGGTTACTACGGCATGGTGTTCGCCATGTTCTCTATAGTCTGTTTAGGTAGGGTAGTATGAGCCCACCATATGTTCTCTATAGGTATGGATGTAAAGACTTCAGTCTTTTTCAGGTCTGTAACTATGATAATTGCGGTACCCACGGGTATCAAGATCTTCACGTGACTTTACATGCTAACCAGAAGCTCAAAAAAGGCCAATAACCCAATCGTGTGGTGGGTGTACGGCTTCATAATACTGTTTACTATCGGAGGG

>32

GCTATTGATGCCATCAATGGTGCTAGTATTCGCTAGTATATGGTTCGGCAGTGGAACAGGATGAACATTTTACCCACCACTGTCCGGGGCCAGGTTTAGTCCAAGTATTGGTACCGACTTTTTGATGTTTTCGCTTCACCTGTCGGGTATTTCTAGTATATTCAGCTCATTAAACTTTATATGTACCATTATAAGAGCTTGAGGTGTGTCCGTGAATATTAAGGACACTGCTATAGTTATATGGGCTTACTTGTTTACGTCCATCTTACTTATATTGTCTCTACCAGTGCTAGCCGCTGGGATAACAATGCTGTTATTTGACCGGAACTTCAACTCATCATTCTTTGACCCAGTGGGCGGAGGGGACCCAGTCCTATTTCAACACTTATTTTGGTTCTTTGGTCACCCAGAGGTGTACGTGCTAATACTCCCGGCATTCGGTATGATTAGTCACATCTGTATAACGTTAAGTAAAGGAGAGCAACCATTTGGTTACTACGGTATGGTGTTCGCCATGTTCTCTATAGTCTGTTTAGGTAGGGTAGTATGAGCCCACCATATGTTCTCTATAGGTATGGATGTAAAGACTTCAGTCTTTTTCAGGTCTGTAACTATGATAATTGCGGTACCCACGGGTATCAAGATCTTCACGTGACTTTACATGCTAGCCAGAAGCTCAAAAAAGGCCAACAACCCAATTGTGTGATGGGTGTACGGCTTCATAATACTGTTTACTATCGGAGGA

>33

GCTATTGATGCCATCAATGGTGCTAGTATTCGCTAGTATATGGTTCGGCAGTGGAACAGGATGAACATTTTACCCACCACTGTCCGGGGCCAGGTTTAGACCAAGTATCGGTACCGACTTTTTGATGTTTTCGCTCCACCTGTCGGGTATTTCTAGTATATTCAGCTCATTAAACTTTATATGCACCATTATAAGAGCTTGAGGTGTGTCCGTGAATATTAAGGACACTGCTATAGTTATATGGGCTTACTTGTTTACGTCCATCTTACTTATATTGTCCCTACCAGTGCTAGCCGCTGGGATAACAATGCTGTTATTTGACCGGAACTTCAACTCATCATTCTTTGACCCAGTGGGCGGAGGGGACCCAGTCCTATTTCAACACTTATTTTGGTTCTTTGGTCACCCAGAGGTGTACGTGCTAATACTCCCGGCATTCGGTATGATTAGTCACATCTGTATAACGCTAAGTAAAGGAGAGCAACCATTTGGTTACTACGGTATGGTGTTCGCCATGTTCTCTATAGTCTGTTTAGGTAGGGTAGTATGAGCCCACCATATGTTCTCTATAGGTATGGATGTAAAGACTTCAGTCTTTTTCAGGTCTGTAACTATGGTAATTGCGGTACCCACGGGTATCAAGATTTTCACGTGACTTTACATGCTAGCCAGAAGCTCAAAAAAGGCCAATAACCCAATCGTGTGGTGGGTGTACGGCTTCATAATACTGTTTACTATCGGAGGA

>34

GCTATTGATGCCATCAATGGTGCTAGTATTCGCTAGTATATGGTTCGGCAGTGGAACAGGATGAACATTTTACCCACCACTGTCCGGGGCCAGGTTTAGACCAAGTATCGGTACCGACTTTTTGATGTTTTCGCTTCACCTGTCGGGTATTTCTAGTATATTCAGCTCATTAAACTTTATATGCACCATTATAAGAGCTTGAGGTGTGTCCGTGAATATTAAGGACACTGCTATAGTTATATGGGCTTACTTGTTTACGTCCATCTTACTCATATTGTCCCTACCAGTGCTAGCCGCTGGGATAACAATGCTGTTATTTGACCGGAACTTCAACTCATCATTCTTTGACCCAGTGGGCGGAGGGGACCCAGTCCTATTTCAACACTTATTTTGGTTCTTTGGTCACCCAGAGGTGTACGTGCTAATACTCCCGGCATTCGGTATGATTAGTCACATCTGTATAACGCTAAGTAAAGGAGAGCAACCATTTGGTTACTACGGTATGGTGTTCGCCATGTTCTCTATAGTCTGTTTAGGTAGGGTAGTATGAGCCCACCATATGTTCTCTATAGGTATGGATGTAAAGACTTCAGTCTTTTTCAGGTCTGTAACTATGGTAATTGCGGTACCCACGGGTATCAAGATCTTCACGTGACTTTACATGCTAGCCAGAAGCTCAAAAAAGGCCAATAACCCAATCGTGTGGTGGGTGTACGGCTTCATAATACTGTTTACTATCGGAGGA

>35

GCTATTAATGCCATCAATGGTGCTAGTATTCGCTAGTATATGGTTCGGCAGTGGAACAGGATGAACATTTTACCCACCACTGTCCGGGGCCAGGTTTAGACCAAGTATCGGTACCGACTTTTTGATGTTTTCGCTTCACCTGTCGGGTATTTCTAGTATATTCAGCTCATTAAACTTTATATGTACCATTATAAGAGCTTGAGGTGTGTCCGTGAATATTAAGGACACTGCTATAGTTATATGGGCTTACTTGTTTACGTCCATCTTACTTATATTGTCCCTACCAGTGCTAGCCGCTGGGATAACAATGCTGTTATTTGACCGGAACTTCAACTCATCCTTCTTTGACCCAGTGGGCGGAGGGGACCCAGTCCTATTTCAACACTTATTTTGGTTCTTTGGTCACCCAGAGGTGTACGTGCTAATACTCCCGGCATTCGGTATGATTAGTCATATCTGTATAACGCTAAGTAAAGGAGAGCAACCATTTGGTTACTACGGCATGGTGTTCGCCATGTTCTCTATAGTCTGTTTAGGTAGGGTAGTATGAGCCCACCATATGTTCTCTATAGGTATGGATGTAAAGACTTCAGTCTTTTTCAGGTCTGTAACTATGGTGATTGCGGTACCCACGGGTATCAAGATCTTCACGTGACTTTACATGCTAGCCAGAAGCTCAAAAAAGGCCAATAACCCAATCGTGTGGTGGGTGTACGGCTTCATAATACTGTTTACTATCGGAGGA

>36

ACTATTGATGCCATCAATGGTGCTAGTATTCGCTAGTATGTGGTTCGGCAGTGGAACAGGATGAACATTTTACCCACCACTGTCCGGGGCCAGGTTTAGTCCAAGCATTGGTACCGACTTTTTGATGTTTTCGCTTCACCTGTCTGGTATTTCTAGTATATTCAGCTCATTAAACTTTATATGCACCATTATAAGAGCTTGAGGTGTGTCCGTGAATATTAAGGACACTGCTATAGTTATATGGGCTTACTTGTTTACGTCCATCTTACTTATATTGTCCCTACCAGTGCTAGCCGCTGGGATAACAATGCTGTTATTTGACCGAAACTTCAACTCATCCTTCTTTGACCCAGTGGGCGGAGGGGACCCAGTCCTATTTCAACACTTATTTTGGTTCTTTGGTCACCCAGAGGTTTACGTGCTAATACTCCCGGCATTCGGTATGATTAGYCACATCTGTATAACGTTAAGTAAAGGAGAGCAACCATTTGGTTACTACGGTATGGTGTTCGCCATGTTCTCTATAGTCTGTTTAGGTAGGGTAGTATGAGCCCACCACATGTTCTCTATAGGTATGGATGTAAAGACTTCAGTCTTTTTCAGGTCTGTAACTATGATAATTGCGGTACCCACGGGCATCAAGATCTTCACGTGACTTTACATGCTAACCAGAAGCTCAAAAAAGGCCAATAACCCAATCGTGTGGTGGGTGTACGGCTTCATAATACTGTTTACTATCGGAGGA

>37

GCTATTGATGCCATCAATGGTGCTAGTATTCGCTAGTATGTGGTTCGGCAGTGGAACAGGATGAACATTTTACCCACCACTGTCCGGGGCCAGGTTTAGTCCAAGCATTGGTACCGACTTTTTGATGTTTTCGCTTCACCTGTCTGGTATTTCTAGTATATTCAGCTCATTAAACTTCATATGCACCATTATAAGAGCTTGAGGTGTGTCCGTGAATATTAAGGACACTGCTATAGTTATATGGGCTTACTTGTTTACGTCCATCTTACTTATATTGTCCCTACCAGTGCTAGCCGCTGGGATAACAATGCTGTTATTTGACCGAAACTTCAACTCATCCTTCTTTGACCCAGTGGGCGGAGGGGACCCAGTCCTATTTCAACACTTATTTTGGTTCTTTGGTCACCCAGAGGTTTACGTGCTAATACTCCCGGCATTCGGTATGATTAGTCACATCTGTATAACGTTAAGTAAAGGAGAGCAACCATTTGGTTACTACGGTATGGTGTTCGCCATGTTCTCTATAGTCTGTTTAGGTAGGGTAGTATGAGCCCACCACATGTTCTCTATAGGTATGGATGTAAAGACTTCAGTCTTTTTCAGGTCTGTAACTATGATAATTGCGGTACCCACGGGCATCAAGATCTTCACGTGACTTTACATGCTAACCAGAAGCTCAAAAAAGGCCAATAACCCAATCGTGTGGTGGGTGTACGGCTTCATAATACTGTTTACTATCGGAGGA

>38

GCTATTGATGCCATCAATGGTGCTAGTATTCGCTAGTATGTGGTTCGGCAGTGGAACAGGATGAACATTTTACCCACCACTGTCCGGGGCCAGGTTTAGTCCAAGCATTGGTACCGACTTTTTGATGTTTTCGCTTCACCTGTCTGGTATTTCTAGTATATTCAGCTCATTAAACTTTATATGCACCATTATAAGAGCTTGAGGTGTGTCCGTGAATATTAAGGACACTGCTATAGTTATATGGGCTTACTTGTTTACGTCCATCTTACTTATATTGTCCCTACCAGTGCTAGCCGCTGGGATAACAATGCTGTTATTTGACCGAAACTTCAACTCATCCTTCTTTGACCCAGTGGGCGGAGGGGACCCAGTCCTATTTCAACACTTATTTTGGTTCTTTGGTCACCCAGAGGTTTACGTGCTAATACTCCCGGCATTCGGTATGATTAGYCACATCTGTATAACGTTAAGTAAAGGAGAGCAACCATTTGGTTACTACGGTATGGTGTTCGCCATGTTCTCTATAGTCTGTTTAGGTAGGGTAGTATGAGCCCACCACATGTTCTCTATAGGTATGGATGTAAAGACTTCAGTCTTTTTCAGGTCTGTAACTATGATAATTGCGGTACCCACGGGCATCAAGATCTTCACGTGACTTTACATGCTAACCAGAAGCTCAAAAAAGGCCAATAACCCAATCGTGTGGTGGGTGTACGGCTTCATAATACTGTTTACTATCGGAGGA

>39

GCTATTGATGCCATCAATGGTGCTAGTATTCGCTAGTATATGGTTCGGCAGTGGAACAGGATGAACATTTTACCCACCACTGTCCGGGGCCAGGTTTAGACCAAGTATCGGTACCGACTTTTTGATGTTTTCGCTCCACCTGTCGGGTATTTCTAGTATATTCAGCTCATTAAACTTTATATGCACCATTATAAGAGCTTGAGGTGTGTCCGTGAATATTAAGGACACTGCTATTGTTATATGGGCTTACTTGTTTACGTCCATCTTACTTATATTGTCCCTACCAGTGCTAGCCGCTGGGATAACAATGCTGTTATTTGACCGGAACTTCAACTCATCATTCTTTGACCCAGTGGGCGGAGGGGACCCAGTCCTATTTCAACACTTATTTTGGTTCTTTGGTCACCCAGAGGTGTACGTGCTAATACTCCCGGCATTCGGTATGATTAGCCACATCTGTATAACGCTAAGTAAAGGAGAGCAACCATTTGGTTACTACGGTATGGTGTTCGCCATGTTCTCTATAGTCTGTTTAGGTAGGGTAGTATGAGCCCACCATATGTTCTCTATAGGTATGGATGTAAAGACTTCAGTCTTTTTCAGGTCTGTAACTATGGTAATTGCGGTACCCACGGGTATCAAGATCTTCACGTGACTTTACATGCTAGCCAGAAGCTCAAAAAAGGCCAATAACCCAATCGTGTGGTGGGTGTACGGCTTCATAATACTGTTTACTATCGGAGGA

>42

GCTATTGATGCCATCAATGGTGCTAGTATTCGCTAGTATATGGTTCGGCAGTGGGACAGGATGAACATTTTACCCACCACTGTCCGGGGCCAGGTTTAGTCCAAGCATTGGTACCGACTTTTTGATGTTTTCGCTTCACCTGTCTGGTATTTCTAGTATATTCAGCTCATTAAACTTTATATGCACCATTATAAGAGCTTGAGGTGTGTCCGTGAATATTAAGGACACTGCTATAGTTATATGGGCTTACTTGTTTACGTCCATCTTACTTATATTGTCCCTACCAGTGCTAGCCGCTGGGATAACAATGCTGTTATTTGACCGGAACTTCAACTCATCATTCTTTGACCCAGTGGGCGGAGGGGACCCAGTCCTATTTCAACACTTATTTTGGTTCTTTGGTCACCCAGAGGTGTACGTGCTAATACTCCCGGCATTCGGTATGATTAGTCACATCTGTATAACGTTAAGTAAAGGAGAGCAACCATTTGGTTACTACGGTATGGTGTTCGCCATGTTCTCTATAGTCTGCTTAGGTAGGGTAGTATGAGCCCACCATATGTTCTCTATAGGTATGGATGTAAAGACTTCAGTCTTTTTCAGGTCTGTAACTATGATAATTGCGGTACCCACGGGTATCAAGATCTTCACGTGACTTTACATGCTAACCAGGAGCTCAAAAAAGGCCAATAACCCAATCGTGTGGTGGGTGTACGGCTTCATAATACTGTTTACTATCGGAGGG

>43

GCTATTGATGCCATCAATGGTGCTAGTATTCGCTAGTATATGGTTCGGCAGTGGGACAGGATGAACATTTTACCCACCACTGTCCGGGGCCAGGTTTAGCCCAAGCATTGGTACCGACTTTTTGATGTTTTCGCTTCACCTGTCTGGTATTTCTAGTATATTCAGCTCATTAAACTTTATATGCACCATTATAAGAGCTTGAGGTGTGTCCGTGAATATTAAGGACACTGCTATAGTTATATGGGCTTACTTGTTTACGTCCATCTTACTTATATTGTCCCTACCAGTGCTAGCCGCTGGGATAACAATGCTGTTATTTGACCGGAACTTCAACTCATCATTCTTTGACCCAGTGGGCGGAGGGGACCCAGTCCTATTTCAACACTTATTTTGGTTCTTTGGTCACCCAGAGGTGTACGTGCTAATACTCCCGGCATTCGGTATGATTAGTCACATCTGTATAACGTTAAGTAAAGGAGAGCAACCATTTGGTTACTACGGTATGGTGTTCGCCATGTTCTCTATAGTCTGCTTAGGTAGGGTAGTATGAGCCCACCATATGTTCTCTATAGGTATGGATGTAAAGACTTCAGTCTTTTTCAGGTCTGTAACTATGATAATTGCGGTACCCACGGGTATCAAGATCTTCACGTGACTTTACATGCTAACCAGGAGCTCAAAAAAGGCCAATAACCCAATCGTGTGGTGGGTGTACGGCTTCATAATACTGTTTACTATCGGAGGG

>44

GCTATTGATGCCATCAATGGTGCTAGTATTCGCTAGTATATGGTTCGGCAGTGGGACAGGATGAACATTTTACCCACCACTGTCCGGGGCCAGGTTTAGTCCAAGCATTGGTACCGACTTTTTGATGTTTTCGCTTCACCTGTCTGGTATTTCTAGTATATTCAGCTCATTAAACTTTATATGCACCATTATAAGAGCTTGAGGTGTGTCCGTGAATATTAAGGACACTGCTATAGTTATATGGGCTTACTTGTTTACGTCCATCTTACTTATATTGTCCCTACCAGTGCTAGCCGCTGGGATAACAATGCTGTTATTTGACCGGAACTTCAACTCATCATTCTTTGACCCAGTGGGCGGAGGGGACCCAGTCCTATTTCAACACTTATTTTGGTTCTTTGGTCACCCAGAGGTGTACGTGCTAATACTCCCGGCATTCGGTATGATTAGTCACATCTGTATAACGTTAAGTAAAGGAGAGCAACCATTTGGTTACTACGGTATGGTGTTCGCCATGTTCTCTATAGTCTGCTTAGGTAGGGTAGTATGAGCCCACCATATGTTCTCTATAGGTATGGATGTAAAGACTTCAGTCTTTTTCAGGTCTGTAACTATGATAATTGCGGTACCCACGGGTATCAAGATCTTCACGTGACTTTACATGCTAACCAGGAGCTCAAAAAAGGCCAGTAACCCAATCGTGTGGTGGGTGTACGGCTTCATAATACTGTTTACTATCGGAGGG

>45

GCTATTGATGCCATCAATGGTGCTAGTATTCGCTAGCATATGGTTCGGCAGTGGGACAGGATGAACATTCTACCCACCACTGTCCGGGGCCAGGTTTAGTCCAAGCATTGGTACCGACTTTTTGATGTTTTCGCTTCACCTGTCTGGTATTTCTAGTATATTCAGCTCATTAAACTTTATATGCACCATTATAAGAGCTTGAGGTGTGTCCGTGAATATTAAGGACACTGCTATAGTTATATGGGCTTACTTGTTTACGTCCATCTTACTTATATTGTCCCTACCAGTGCTAGCCGCTGGGATAACAATGCTGTTATTTGACCGGAACTTCAACTCATCATTCTTTGACCCAGTGGGCGGAGGGGACCCAGTCCTATTTCAACACTTATTTTGGTTCTTTGGTCACCCAGAGGTGTACGTGCTAATACTCCCGGCATTCGGTATGATTAGTCACATCTGTATAACGTTAAGTAAAGGAGAGCAACCATTTGGTTACTACGGTATGGTGTTCGCCATGTTCTCTATAGTCTGCTTAGGTAGGGTAGTATGAGCCCACCATATGTTCTCTATAGGTATGGATGTAAAGACTTCAGTCTTTTTCAGGTCTGTAACTATGATAATTGCGGTGCCCACGGGTATCAAGATCTTCACGTGGCTTTACATGCTAACCAGAAGCTCAAAAAAGGCCAATAACCCAATCGTGTGGTGGGTGTACGGGTTCATAATACTGTTTACTATCGGAGGA

>46

GCTATTGATGCCATCAATGGTGCTAGTATTCGCTAGTATATGGTTCGGCAGTGGAACAGGATGAACATTTTACCCACCACTGTCCGGGGCCAGGTTTAGTCCAAGCATTGGTACCGACTTTTTGATGTTTTCGCTTCACCTGTCTGGTATTTCTAGTATATTCAGCTCATTAAACTTTATATGCACCATTATAAGAGCTTGAGGTGTGTCCGTGAATATTAAGGACACTGCTATAGTTATATGGGCTTACTTGTTTACGTCCATCTTACTTATATTGTCCCTACCAGTGCTAGCCGCTGGGATAACAATGCTGTTATTTGACCGGAACTTCAACTCATCATTCTTTGACCCAGTGGGCGGAGGGGACCCAGTCCTATTTCAACACTTATTTTGGTTCTTTGGCCACCCAGAGGTGTACGTGCTAATACTCCCGGCATTCGGTATGATTAGTCACATCTGTATAACGTTAAGTAAAGGAGAGCAACCATTTGGTTACTACGGTATGGTGTTCGCCATGTTCTCTATAGTCTGTTTAGGTAGGGTAGTATGAGCCCACCATATGTTCTCTATAGGTATGGATGTAAAGACTTCAGTCTTTTTCAGGTCTGTAACTATGATAATTGCGGTACCCACGGGTATCAAGATCTTCACGTGACTTTACATGCTAACCAGAAGCTCAAAAAAGGCCAATAACCCAATCGTGTGGTGGGTGTACGGCTTCATAATACTGTTTACTATCGGAGGA

>47

GCTATTGATGCCATCAATGGTGCTAGTATTCGCTAGTATATGGTTCGGCAGTGGAACAGGATGAACATTTTACCCACCACTGTCCGGGGCCAGGTTTAGTCCAAGCATTGGTACCGACTTTTTGATGTTTTCGCTTCACCTGTCTGGTATTTCTAGTATATTCAGCTCATTAAACTTTATATGCACCATTATAAGAGCTTGAGGTGTGTCCGTGAATATTAAGGACACTGCTATAGTTATATGGGCTTACTTGTTTACGTCCATCTTACTTATATTGTCCCTACCAGTGCTAGCCGCTGGGATAACAATGCTGTTATTTGACCGGAACTTCAACTCATCATTCTTTGACCCAGTGGGCGGAGGGGACCCATTCCTATTTCAACACTTATTTTGGTTCTTTGGTCACCCAGAGGTGTACGTGCTAATACTCCCGGCATTCGGTATGATTAGTCACATCTGTATAACGTTAAGTAAAGGAGAGCAACCATTTGGTTACTACGGTATGGTGTTCGCCATGTTCTCTATAGTCTGTTTAGGTAGGGTAGTATGAGCCCACCATATGTTCTCTATAGGTATGGATGTAAAGACTTCAGTCTTTTTCAGGTCTGTAACTATGATAATTGCGGTACCCACGGGTATCAAGATCTTCACGTGACTTTACATGCTAACCAGAAGCTCAAAAAAGGCCAATAACCCAATCGTGTGGTGGGTGTACGGCTTCATAATACTGTTTACTATCGGAGGA

>48

GCTATTGATGCCATCAATGGTGCTAGTATTCGCTAGTATGTGGTTCGGCAGTGGAACAGGATGAACATTTTACCCACCACTGTCCGGGGCCAGGTTTAGTCCAAGCATTGGTACCGACTTTTTGATGTTTTCGCTTCACCTGTCTGGTATTTCTAGTATATTCAGCTCATTAAACTTTATATGCACCATTATAAGAGCTTGAGGTGTGTCCGTGAATATTAAGGACACTGCTATAGTTATATGGGCTTACTTGTTTACGTCCATCTTACTTATATTGTCCCTACCAGTGCTAGCCGCTGGGATAACAATGCTGTTATTTGACCGGAACTTCAACTCATCATTCTTTGACCCAGTGGGCGGGGGGGACCCAGTCCTATTTCAACACTTATTTTGGTTCTTTGGCCACCCAGAGGTGTACGTGCTAATACTCCCGGCATTCGGTATGATTAGTCACATCTGTATAACGTTAAGCAAAGGAGAGCAACCATTTGGTTACTACGGTATGGTGTTCGCCATGTTCTCTATAGTCTGTTTAGGTAGGGTAGTATGAGCCCACCATATGTTCTCTATAGGTATGGATGTAAAGACTTCAGTCTTTTTCAGGTCTGTAACTATGATAATTGCGGTACCCACGGGTATCAAGATCTTCACGTGACTTTACATGCTAACCAGAAGCTCAAAAAAGGCCAATAACCCAATCGTGTGGTGGGTGTACGGCTTCATAATACTGTTTACTATCGGAGGA

>49

GCTATTGATGCCATCAATGGTGCTAGTATTCGCTAGTATATGGTTCGGCAGTGGAACAGGATGAACATTTTACCCACCACTGTCCGGGGCCAGGTTTAGACCAAGTATCGGTACCGACTTTTTGATGTTTTCGCTCCACCTGTCGGGTATTTCTAGTATATTCAGCTCATTAAACTTTATATGCACCATTATAAGAGCTTGAGGTGTGTCCGTGAATATTAAGGACACTGCTATTGTTATATGGGCTTACTTGTTTACGTCCATCTTACTTATATTGTCCCTACCAGTGCTAGCCGCTGGGATAACAATGCTGTTATTCGACCGGAACTTCAACTCATCATTCTTTGACCCAGTGGGCGGAGGGGACCCAGTCCTATTTCAACACTTATTTTGGTTCTTTGGTCACCCAGAGGTGTACGTGCTAATACTCCCGGCATTCGGTATGATTAGTCACATCTGTATAACGCTAAGTAAAGGAGAGCAACCATTTGGTTACTACGGTATGGTGTTCGCCATGTTCTCTATAGTCTGTTTAGGTAGGGTAGTATGAGCCCACCATATGTTCTCTATAGGTATGGATGTAAAGACTTCAGTCTTTTTCAGGTCTGTAACTATGGTAATTGCGGTACCCACGGGTATCAAGATCTTCACGTGACTTTACATGCTAGCCAGAAGCTCAAAAAAGGCCAATAACCCAATCGTGTGGTGGGTGTACGGCTTCATAATACTGTTTACTATCGGAGGA

>50

ATTACTAATGCCGTCAATGGTGTTAGTATTCGCTAGTATATGGTTTGGTAGAGGAACAGGATGAACTTTCTACCCCCCATTATCAGGGGCTAGGTTCAGCCCAAGAGTTGGTACAGACTTCTTAATGTTTTCTCTTCACTTATCTGGTATATCTAGCATATTTAGGTCACTTAATTTTATATGTACTATAATAAGCGCATGAGGTGTCTCAGTAAATGTTAAGGACACAGCAATAGTAATATGAGCATACTTATTTACCTCTATATTATTAATACTTTCTTTACCAGTGTTAGCAGCGGGAATTACAATGCTATTGTTTGATCGTAAATTCAACTCATCATTCTTCGACCCAGTGGGGGGAGGTGATCCAGTTCTGTTTCAACATCTATTCTGGTTCTTTGGACACCCAGAAGTGTATGTACTAATCCTACCAGCATTTGGTATGATTAGTCATATATGTATTACACTAAGAAACGGTGAACAACCGTTTGGTTATTACGGAATGGTGTTTGCCATGTTTTCAATAGTGTGCCTCGGTAGAGTAGTCTGAGCTCACCATATGTTTTCTATAGGTATGGATGTAAAGACTTCAGTGTTCTTTAGATCAGTAACAATGATAATAGCTGTTCCAACAGGAATAAAGATATTTACCTGACTATACATGTTATCTAGAAGATCAAACAAGTTCAATAACCCAATAGTATGATGGGTATACGGATTTATAATTTTATTTACTATAGGTGGT

>51

GCTATTGATGCCATCAATGGTGCTAGTATTCGCTAGTATATGGTTCGGCAGTGGAACAGGATGAACTTTTTACCCACCACTGTCCGGGGCCAGGTTTAGTCCAAGCATTGGTACCGACTTTTTGATGTTTTCGCTTCACCTGTCGGGTATTTCTAGTATATTCAGCTCATTAAACTTTATATGCACCATTATAAGGGCTTGAGGTGTGTCCGTGAATATTAAGGACACTGCTATAGTTATATGGGCTTACTTGTTTACGTCCATCTTACTTATATTGTCCCTACCAGTGCTAGCCGCTGGGATAACAATGTTGTTATTTGACCGGAACTTCAATTCATCATTCTTTGATCCAGTGGGCGGAGGGGACCCAGTCCTATTTCAACACTTATTTTGGTTCTTTGGTCACCCAGAGGTGTACGTGCTAATACTCCCGGCATTCGGTATGATTAGTCACATCTGTATAACGTTAAGTAAAGGAGAGCAACCATTTGGTTACTACGGTATGGTGTTCGCCATGTTCTCTATAGTCTGTTTAGGTAGGGTAGTATGGGCCCACCATATGTTCTCTATAGGTATGGATGTAAAGACTTCAGTCTTTTTCAGGTCTGTAACTATGATAATTGCGGTACCCACGGGTATCAAGATCTTCACGTGGCTTTACATGCTAACCAGAAGCTCAAAAAAGGCCAATAACCCAATCGTGTGGTGGGTATACGGCTTCATAATACTATTTACTATCGGAGGA

>52

GCTATTGATGCCATCAATGGTGCTAGTATTCGCTAGTATATGGTTCGGCAGTGGAACAGGATGAACATTTTACCCCCCACTGTCCGGGGCCAGGTTTAGTCCAAGTATTGGTACCGACTTTTTGATGTTTTCGCTTCACCTGTCTGGTATTTCTAGTATATTCAGCTCATTAAACTTCATATGCACCATTATAAGGGCTTGAGGTGTGTCCGTGAATATTAAGGACACTGCTATAGTTATATGGGCTTACTTGTTTACGTCCATCTTACTTATATTGTCCCTACCAGTGCTAGCTGCTGGGATAACAATGCTGTTATTTGACCGGAACTTCAACTCATCATTCTTTGACCCAGTGGGTGGAGGGGACCCAGTCCTATTTCAACACCTATTTTGGTTCTTTGGTCACCCAGAGGTGTACGTGCTAATACTCCCGGCATTCGGTATGATTAGCCACATCTGTATAACGCTAAGTAAAGGAGAGCAACCATTTGGTTACTACGGTATGGTGTTCGCCATGTTCTCTATAGTCTGCTTAGGTAGGGTAGTATGAGCCCATCATATGTTCTCTATAGGTATGGATGTAAAGACTTCAGTCTTTTTCAGATCTGTAACTATGATAATTGCGGTACCCACGGGTATCAAGATCTTCACGTGACTTTACATGCTAACCAGAAGCTCAAAAAAGGCCAATAACCCAATCGTGTGGTGGGTGTACGGCTTCATAATACTGTTCACTATCGGAGGA

>53

GCTATTGATGCCATCAATGGTGCTAGTATTCGCTAGTATATGGTTCGGCAGTGGAACAGGATGAACTTTTTACCCACCACTGTCCGGGGCCAGGTTTAGTCCAAGCATTGGTACCGACTTTTTGATGTTTTCGCTTCACCTGTCGGGTATTTCTAGTATATTCAGCTCATTAAACTTTATATGTACCATTATAAGGGCTTGAGGTGTGTCCGTGAATATTAAGGACACTGCTATAGTTATATGGGCTTACTTGTTTACGTCCATCTTACTTATATTGTCCCTACCAGTGCTAGCCGCTGGGATAACAATGTTGTTATTTGACCGGAACTTCAATTCATCATTCTTTGACCCAGTGGGCGGAGGGGACCCAGTCCTATTTCAACACTTATTTTGGTTCTTTGGTCACCCAGAGGTGTACGTGCTAATACTCCCGGCATTCGGTATGATTAGTCACATCTGTATAACGTTAAGTAAAGGAGAGCAACCATTTGGTTACTACGGTATGGTGTTCGCCATGTTCTCTATAGTCTGTTTAGGTAGGGTAGTATGGGCCCACCATATGTTCTCTATAGGTATGGATGTAAAGACTTCAGTCTTTTTCAGGTCTGTAACTATGATAATTGCGGTACCCACGGGTATCAAGATCTTCACGTGGCTTTACATGCTAACCAGAAGCTCAAAAAAGGCCAATAACCCAATCGTGTGGTGGGTATACGGCTTCATAATACTATTTACTATCGGAGGA

>54

GCTATTGATGCCATCAATGGTGCTAGTATTCGCTAGTATATGGTTCGGCAGTGGAACAGGATGAACTTTTTACCCACCACTGTCCGGGGCCAGGTTTAGTCCAAGCATTGGTACCGACTTTTTGATGTTTTCGCTTCACCTGTCGGGTATTTCTAGTATATTCAGCTCATTAAACTTTATATGCACCATTATAAGGGCTTGAGGTGTGTCCGTGAATATTAAGGACACTGCTATAGTTATATGGGCTTACTTGTTTACGTCCATCTTACTTATATTGTCCCTACCAGTGCTAGCCGCTGGGATAACAATGTTGTTATTTGACCGGAACTTCAATTCATCATTCTTTGATCCAGTGGGCGGAGGGGACCCAGTCCTATTTCAACACTTATTTTGGTTCTTTGGTCACCCAGAGGTGTACGTGCTAATACTCCCGGCATTCGGTATGATTAGTCACATCTGTATAACGTTAAGTAAAGGAGAGCAACCATTTGGTTACTACGGTATGGTGTTCGCCATGTTCTCTATAGTCTGTTTAGGTAGGGTAGTATGGGCCCACCATATGTTCTCTATAGGTATGGATGTAAAGACTTCAGTCTTTTTCAGGTCTGTAACTATGATAATTGCGGTACCCACGGGTATCAAGATCTTCACGTGGCTTTACATGCTAACCAGAAGCTCAAAAAAGACCAATAACCCAATCGTGTGGTGGGTATACGGCTTCATAATACTATTTACTATCGGAGGA

>55

GCTATTGATGCCATCAATGGTGCTAGTATTCGCTAGTATATGGTTCGGCAGTGGAACAGGATGAACATTTTACCCACCACTGTCCGGGGCCAGGTTTAGTCCAAGCATTGGTACCGACTTTTTGATGTTTTCGCTTCACCTGTCGGGTATTTCTAGTATATTCAGCTCATTAAACTTTATATGCACCATTATAAGGGCTTGAGGTGTGTCCGTGAATATTAAGGACACTGCTATAGTTATATGGGCTTACTTGTTTACGTCCATCTTACTTATATTGTCCCTACCAGTGCTAGCCGCTGGGATAACAATGTTGTTATTTGACCGGAACTTCAATTCATCATTCTTTGACCCAGTGGGCGGAGGGGACCCAGTCCTATTTCAACACTTATTTTGGTTCTTTGGTCACCCAGAGGTGTACGTGCTAATACTCCCGGCATTCGGTATGATTAGTCACATCTGTATAACGTTAAGTAAAGGAGAGCAACCATTTGGTTACTACGGTATGGTGTTCGCCATGTTCTCTATAGTCTGTTTAGGTAGGGTAGTATGGGCCCACCATATGTTCTCTATAGGTATGGATGTAAAGACTTCAGTCTTTTTCAGGTCTGTAACTATGATAATTGCGGTACCCACGGGTATCAAGATCTTCACGTGGCTTTACCTGCTAACCAGAAGCTCAAAAAAGGCCAATAACCCAATCGTGTGGTGGGTATACGGCTTCATAATATTATTTACTATCGGAGGA

>56

GCTATTGATGCCATCAATGGTGCTAGTATTCGCTAGTATATGGTTCGGCAGTGGAACAGGATGAACATTTTACCCACCACTGTCCGGGGCCAGGTTTAGTCCAAGCATTGGTACCGACTTTTTGATGTTTTCGCTTCACCTGTCTGGTATTTCTAGTATATTCAGCTCATTAAACTTTATATGCACCATTATAAGGGCTTGAGGTGTGTCCGTGAATATTAAGGACACTGCTATAGTTATATGGGCTTACTTGTTTACGTCCATCTTACTTATATTGTCCCTACCAGTGCTAGCCGCTGGGATAACAATGTTGTTATTTGACCGGAACTTCAACTCATCATTCTTTGACCCAGTGGGCGGAGGGGACCCAGTCCTATTTCAACACTTATTTTGGTTCTTTGGTCACCCAGAGGTGTACGTGCTAATACTCCCGGCATTCGGTATGATTAGTCACATCTGTATAACGTTAAGTAAAGGAGAGCAACCATTTGGTTACTACGGTATGGTGTTCGCCATGTTCTCTATAGTCTGTTTAGGTAGGGTAGTATGGGCCCACCATATGTTCTCTATAGGTATGGATGTAAAGACTTCAGTCTTTTTCAGGTCTGTAACTATGATAATTGCGGTACCCACGGGTATCAAGATCTTCACGTGGCTTTACATGCTAACCAGAAGCTCAAAAAAGGCCAATAACCCAATCGTGTGGTGGGTATACGGCTTCATAATACTATTTACTATCGGAGGA

>57

GCTATTGATGCCATCAATGGTGCTAGTATTCGCTAGTATGTGGTTCGGCAGTGGAACGGGATGAACATTTTACCCACCACTGTCCGGGGCCAGGTTTAGTCCAAGCATTGGTACCGACTTTTTGATGTTTTCGCTTCACCTGTCTGGTATTTCTAGTATATTCAGCTCATTAAACTTTATATGTACTATTATAAGAGCTTGAGGTGTGTCCGTGAATATTAAGGACACTGCTATAGTTATATGGGCTTACTTGTTTACGTCCATCTTACTTATATTGTCCCTACCAGTGCTAGCCGCTGGGATAACAATGCTGCTATTTGATCGGAACTTCAACTCATCATTCTTTGACCCAGTGGGTGGAGGGGACCCAGTCCTATTTCAACACTTATTTTGGTTCTTTGGTCACCCAGAGGTGTACGTGCTGATACTCCCGGCATTCGGTATGATTAGTCACATCTGTATAACGTTAAGTAAAGGAGAGCAGCCATTTGGTTACTACGGTATGGTGTTCGCCATGTTCTCTATAGTCTGTTTAGGTAGGGTAGTATGAGCCCACCATATGTTCTCTATAGGTATGGATGTAAAGACTTCAGTCTTTTTCAGGTCTGTAACTATGATAATTGCGGTACCCACGGGTATCAAGATCTTCACGTGACTTTACATGCTAACCAGAAGCTCAAAAAAGGCCAATAACCCAATCGTGTGGTGGGTATACGGCTTCATAATACTATTCACTATCGGAGGA

>58

GCTATTGATGCCATCAATGGTGCTAGTATTCGCCAGTATGTGGTTCGGCAGTGGAACAGGATGAACATTTTACCCACCACTGTCCGGGGCCAGGTTTAGTCCAAGCATTGGTACCGACTTTTTGATGTTTTCGCTTCACCTGTCTGGTATTTCTAGTATATTCAGCTCATTAAACTTTATATGCACCATTATAAGAGCTTGAGGTGTGTCCGTGAATATTAAGGACACTGCTATAGTTATATGGGCTTACTTGTTTACGTCCATCTTACTTATATTGTCCCTACCAGTGCTAGCCGCTGGGATAACAATGCTGTTATTTGACCGGAACTTCAACTCATCATTCTTTGACCCAGTGGGCGGAGGGGACCCAGTCCTATTTCAACACTTATTTTGGTTCTTTGGTCACCCAGAAGTGTACGTGCTAATACTCCCGGCATTCGGCATGATTAGTCACATCTGTATAACGTTAAGTAAAGGAGAGCAACCATTTGGTTACTACGGTATGGTGTTCGCCATGTTCTCTATAGTCTGTTTAGGTAGGGTAGTATGAGCCCACCATATGTTCTCTATAGGTATGGATGTAAAGACTTCAGTCTTTTTCAGGTCTGTAACTATGATAATTGCGGTACCCACGGGCATCAAGATCTTCACGTGACTTTACATGCTAACCAGAAGCTCAAAAAAGGCCAATAACCCAATCGTGTGGTGGGTATACGGCTTCATAATACTGTTTACTATCGGAGGA

>59

GCTATTGATGCCATCAATGGTGCTAGTATTCGCTAGTATGTGGTTCGGCAGTGGAACAGGATGAACATTTTACCCACCACTGTCCGGGGCCAGGTTTAGTACAAGCATTGGTACCGACTTTTTGATGTTTTCGCTTCACCTGTCTGGTATTTCTAGTATATTCAGCTCATTAAACTTTATATGCACCATTATAAGAGCTTGAGGTGTGTCCGTGAATATTAAGGATACTGCTATAGTTATATGGGCTTACTTGTTTACGTCCATCTTACTTATATTGTCCCTACCAGTGTTAGCCGCTGGGATAACAATGCTGTTATTTGACCGGAACTTCAACTCATCATTCTTTGACCCAGTGGGTGGAGGGGACCCAGTCTTATTTCAACACTTATTTTGGTTCTTCGGTCACCCAGAGGTGTACGTGCTAATACTTCCGGCATTCGGTATGATTAGTCACATCTGTATAACGCTAAGTAAAGGAGAGCAACCATTTGGTTACTACGGTATGGTGTTCGCCATGTTCTCTATAGTCTGTTTAGGTAGGGTAGTATGAGCCCATCATATGTTCTCTATAGGTATGGATGTGAAGACTTCAGTCTTTTTCAGGTCTGTAACTATGATAATCGCGGTACCCACGGGTATCAAGATCTTCACGTGACTTTACATGCTAACCAGAAGCTCAAAAAAGGCCAATAACCCAATCGTGTGGTGGGTGTACGGCTTCATAATACTGTTTACTATCGGAGGA

>60

GCTATTGATGCCATCAATGGTGCTAGTATTCGCTAGTATGTGGTTCGGCAGTGGAACGGGATGAACATTTTACCCACCACTGTCCGGGGCCAGGTTTAGTCCAAGCATTGGTACCGACTTTTTGATGTTTTCGCTTCACCTGTCTGGTATTTCTAGTATATTCAGCTCATTAAACTTTATATGTACCATTATAAGAGCTTGAGGTGTGTCCGTGAATATTAAGGACACTGCTATAGTTATATGGGCTTACTTGTTTACGTCCATCTTACTTATATTGTCCCTACCAGTGCTAGCCGCTGGGATAACAATGCTGCTATTTGATCGGAACTTCAACTCATCATTCTTTGACCCAGTGGGTGGAGGGGACCCAGTCCTATTTCAACACTTATTTTGGTTCTTTGGTCACCCAGAGGTGTACGTGCTAATACTCCCGGCATTCGGTATGATCAGTCACATCTGTATAACGTTAAGTAAAGGAGAGCAACCATTTGGTTACTACGGTATGGTGTTCGCCATGTTCTCTATAGTCTGTTTAGGTAGGGTAGTATGAGCCCACCATATGTTCTCTATAGGTATGGATGTAAAGACTTCAGTCTTTTTCAGGTCTGTAACTATGATAATTGCGGTACCCACGGGTATCAAGATCTTCACGTGACTTTACATGCTAACCAGAAGCTCAAAAAAGGCCAATAACCCAATCGTGTGGTGGGTATACGGCTTCATAATACTATTCACTATCGGAGGA

>61

GCTATTGATGCCATCAATGGTGCTAGTATTCGCTAGTATATGGTTCGGCAGTGGAACAGGATGAACATTTTACCCACCACTGTCCGGGGCCAGGTTTAGTCCAAGCATTGGTACCGACTTTTTAATGTTTTCGCTTCACCTGTCTGGTATTTCTAGTATATTCAGGTCATTAAACTTTATATGCACCATTATAAGAGCTTGAGGTGTGTCCGTGAATATTAAGGACACTGCTATAGTCATATGGGCTTACTTGTTTACGTCCATCTTACTTATATTGTCCCTACCAGTGCTAGCCGCTGGGATAACAATGCTGTTATTTGACCGGAACTTCAACTCATCATTCTTTGACCCAGTGGGTGGAGGGGATCCAGTCCTATTTCAACACTTATTTTGGTTCTTTGGTCACCCAGAGGTGTACGTGCTAATACTCCCGGCATTCGGTATGATTAGTCACATTTGTATAACTTTAAGTAAAGGAGAGCAACCATTTGGTTACTACGGTATGGTGTTCGCCATGTTCTCTATAGTCTGTTTAGGTAGGGTAGTGTGGGCCCACCATATGTTCTCTATAGGTATGGATGTAAAGACTTCAGTCTTTTTCAGGTCTGTAACTATGATAATTGCGGTACCCACGGGTATCAAGATCTTCACGTGACTTTACATGCTAACCAGTAGCTCAAAAAAGGCCAATAACCCAATCGTGTGGTGGGTGTACGGCTTCATAATACTGTTTACTATCGGAGGA

>62

GCTATTGATGCCATCAATGGTGCTAGTATTCGCTAGTATATGGTTCGGCAGTGGAACAGGATGAACATTTTACCCACCACTGTCCGGGGCCAGGTTTAGTCCAAGCGTTGGTACCGACTTTTTGATGTTTTCGCTTCACCTGTCTGGTATCTCTAGTATATTCAGCTCATTAAACTTTATATGCACCATTATAAGAGCTTGAGGTGTGTCCGTGAATATTAAGGACACTGCTATAGTTATATGGGCTTACTTGTTTACGTCCATCTTGCTTATACTGTCCCTACCAGTGCTAGCCGCTGGGATAACAATGCTGTTATTTGACCGGAACTTCAACTCATCATTCTTTGACCCAGTGGGCGGAGGGGACCCAGTCCTATTTCAACACTTATTTTGGTTCTTTGGTCACCCAGAGGTGTACGTGCTAATACTCCCGGCATTCGGTATGGTTAGTCACATCTGTATAACGCTAAGTAAAGGAGAGCAACCATTTGGTTACTACGGCATGGTGTTCGCCATGTTCTCTATAGTCTGTTTAGGTAGGGTAGTATGAGCCCACCATATGTTCTCTATAGGTATGGATGTAAAGACTTCAGTCTTTTTCAGGTCTGTAACTATGATAATTGCGGTACCCACGGGTATCAAGATCTTTACGTGACTTTACATGCTAACCAGAAGCTCAAAAAAGGCCAATAACCCAATCGTGTGGTGGGTGTACGGCTTCATAATACTGTTTACTATCGGAGGA

>63

GCTATTGATGCCATCAATGGTGCTAGTATTCGCTAGTATGTGGTTCGGCAGTGGAACAGGATGAACATTTTACCCACCACTGTCCGGGGCCAGGTTTAGTCCAAGTATTGGTACCGACTTTTTGATGTTTTCGCTTCACCTGTCTGGTATTTCTAGTATATTCAGCTCATTAAACTTTATATGTACCATTATAAGAGCTTGAGGTGTGTCCGTGAGTATTAAGGACACTGCTATAGTTATATGGGCTTACTTGTTTACGTCCATCTTACTTATATTGTCCCTACCAGTGCTAGCCGCTGGGATAACAATGCTGTTATTTGACCGGAACTTCAACTCATCATTCTTTGACCCAGTGGGCGGAGGAGACCCTGTCTTATTTCAACACTTATTTTGGTTCTTTGGTCACCCAGAGGTGTACGTGCTAATACTCCCGGCATTCGGCATGATTAGTCACATCTGTATAACGTTAAGTAAAGGAGAGCAACCATTTGGTTACTACGGTATGGTGTTCGCCATGTTCTCTATAGTCTGTTTAGGTAGGGTAGTATGGGCCCACCATATGTTCTCTATAGGTATGGATGTAAAGACTTCAGTCTTTTTCAGGTCTGTAACTATGGTAATTGCGGTACCCACGGGTATCAAGATCTTCACGTGACTTTACATGCTAGCCAGAAGCTCAAAAAAGGCCAATAACCCAATCGTGTGGTGGGTGTACGGCTTCATAATACTGTTTACTATCGGAGGA

>64

GCTATTGATGCCATCAATGGTGCTAGTATTCGCTAGTATATGGTTCGGCAGTGGAACAGGATGAACATTTTACCCACCACTGTCCGGGGCCAGGTTTAGTCCAAGCATTGGTACCGACTTTTTGATGTTTTCGCTTCACCTGTCTGGTATTTCTAGTATATTCAGCTCATTAAACTTTATATGCACCATTATAAGGGCTTGAGGTGTGTCCGTGAATATTAAGGACACTGCTATAGTTATATGGGCTTACTTGTTTACGTCCATCTTACTTATATTGTCCCTACCAGTGCTAGCCGCTGGGATAACAATGTTGTTATTTGACCGGAACTTCAACTCATCATTCTTTGACCCAGTGGGCGGAGGGGACCCAGTCCTATTTCAACACTTATTTTGGTTCTTTGGTCACCCAGAGGTGTACGTGCTAATACTCCCGGCATTCGGTATGATTAGTCACATCTGTATAACGTTAAGTAAAGGAGAGCAACCATTTGGTTACTACGGTATGGTGTTCGCCATGTTCTCTATAGTCTGTTTAGGTAGGGTAGTATGGGCCCACCATATGTTCTCTATAGGTATGGATGTAAAGACTTCAGTCTTTTTCAGGTCTGTAACTATGATAATTGCGGTACCCACGGGTATCAAGATCTTCACGTGGCTTTACATGCTAACCAGAAGCTCAAAAAAGGCCAATAACCCAATCGTGTGGTGGGTATACGGATTCATAATACTATTTACTATCGGAGGA
